# Reticular chemistry guided precise construction of zirconium-pentacarboxylate frameworks with 5-connected Zr_6_ clusters†

**DOI:** 10.1039/d3sc05410a

**Published:** 2024-01-23

**Authors:** Tianyou Peng, Chao-Qin Han, Hai-Lun Xia, Kang Zhou, Jian Zhang, Jincheng Si, Lei Wang, Jiafeng Miao, Fu-An Guo, Hao Wang, Lu-Lu Qu, Guozhong Xu, Jing Li, Xiao-Yuan Liu

**Affiliations:** a Hoffmann Institute of Advanced Materials, Shenzhen Polytechnic University 7098 Liuxian Blvd, Nanshan District Shenzhen 518055 P. R. China liuxiaoyuan1989@szpu.edu.cn jingli@rutgers.edu; b College of Chemical Engineering, University of Science and Technology Liaoning Anshan 114051 P. R. China gz_xu@ustl.edu.cn; c School of Chemistry and Materials Science, Jiangsu Normal University Xuzhou 221116 P. R. China; d Department of Chemistry and Chemical Biology, Rutgers University 123 Bevier Road Piscataway New Jersey 08854 USA

## Abstract

Zirconium-based metal–organic frameworks (Zr-MOFs) have been extensively studied due to their very rich structural chemistry. The combination of nearly unlimited carboxylic acid-based linkers and Zr_6_ clusters with multiple connectivities has led to diverse structures and specific properties of resultant Zr-MOFs. Herein, we demonstrate the successful use of reticular chemistry to construct two novel Zr-MOFs, HIAM-4040 and HIAM-4040-OH, with zfu topology. Based on a thorough structural analysis of (4,4)-connected lvt-type Zr-tetracarboxylate frameworks and a judicious linker design, we have obtained the first example of a Zr-pentacarboxylate framework featuring unprecedented 5-connected organic linkers and 5-connected Zr_6_ clusters. Compared with HIAM-4040, a larger Stokes shift is achieved in HIAM-4040-OH *via* hydroxyl group induced excited-state intramolecular proton transfer (ESIPT). HIAM-4040-OH exhibits high chemical and thermal stability and is used for HClO detection in aqueous solution with excellent sensitivity and selectivity.

## Abstract 2

A zirconium-pentacarboxylate framework is constructed using a pentacarboxylic acid with 5-connected Zr6 clusters guided by reticular chemistry. One isoreticular MOF can be utilized for HClO detection with excellent sensitivity and selectivity.

## Graphical abstract

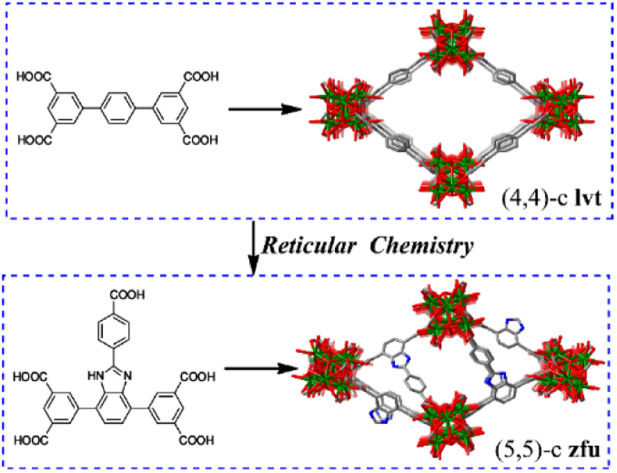

## Introduction

As one of the most extensively studied subclasses, zirconium-based metal–organic frameworks (Zr-MOFs)^1–6^ possess rich structural diversity and functionalizable sites, outstanding chemical and thermal stability, and intriguing properties. They are considered to be one of the most promising MOF branches for practical applications. The tunable linker length, number of carboxylate groups, geometry and functionality of organic linkers, and variable connection numbers of Zr_6_ clusters not only provide the structural abundance of Zr-MOFs, but also generate specific properties. Up to now, Zr-MOFs made of ditopic,^7–10^ tritopic,^11–15^ tetratopic^16–21^ and hexatopic^22–24^ carboxylic acids have been reported with 3-,^25^ 4-,^19,26^ 6-,^22^ 8-,^16,17,21^ 9-,^15,27^ 10-^28^ or 12-connected^7,10,18^ Zr_6_ clusters and different underlying nets. Benefiting from the aforementioned advantages, Zr-MOFs have exhibited excellent potential for various applications, including but not limited to catalysis,^15,16,29^ chemical storage and separation,^19,30^ sensing^6,13,31^ and imaging.^32–34^

While many Zr_6_-based MOFs have been synthesized since their first discovery in 2008,^7^ those with 5-, 7- and 11-connected Zr_6_ clusters have rarely been reported. Likewise, although di-, tri-, tetra- and hexa-topic linkers have all been successfully used in constructing Zr-MOFs, pentacarboxylate-based structures do not exist to this date. Designing and synthesizing suitable pentacarboxylic acids to obtain corresponding Zr-MOFs have been found to be very difficult although several pentacarboxylic acids have been reported to have been used to construct Co- or Cu-MOFs more than 10 years ago.^5,35,36^ This fact might be ascribed to the geometric frustration as reported, in which shape-mismatched linkers will prohibit the formation of predetermined frameworks.^37^ Fortunately, the establishment and development of reticular chemistry and the Reticular Chemistry Structure Resource (RCSR) database have been proven to be useful approaches in guiding the construction of novel MOF structures *via* the top-down design and precious assembly of predesigned inorganic building units and organic linkers at the molecular level.^38–45^ Reticular chemistry has been proven to be a useful strategy in designing many structure-^9,22,46,47^ and property-specific^48,49^ MOFs. Therefore, we envision that it can also help guide the development of pentacarboxylate-based Zr-MOFs and the formation of 5-, 7-, and 11-connected Zr_6_ cluster-based MOFs.

**Fig. 1:**
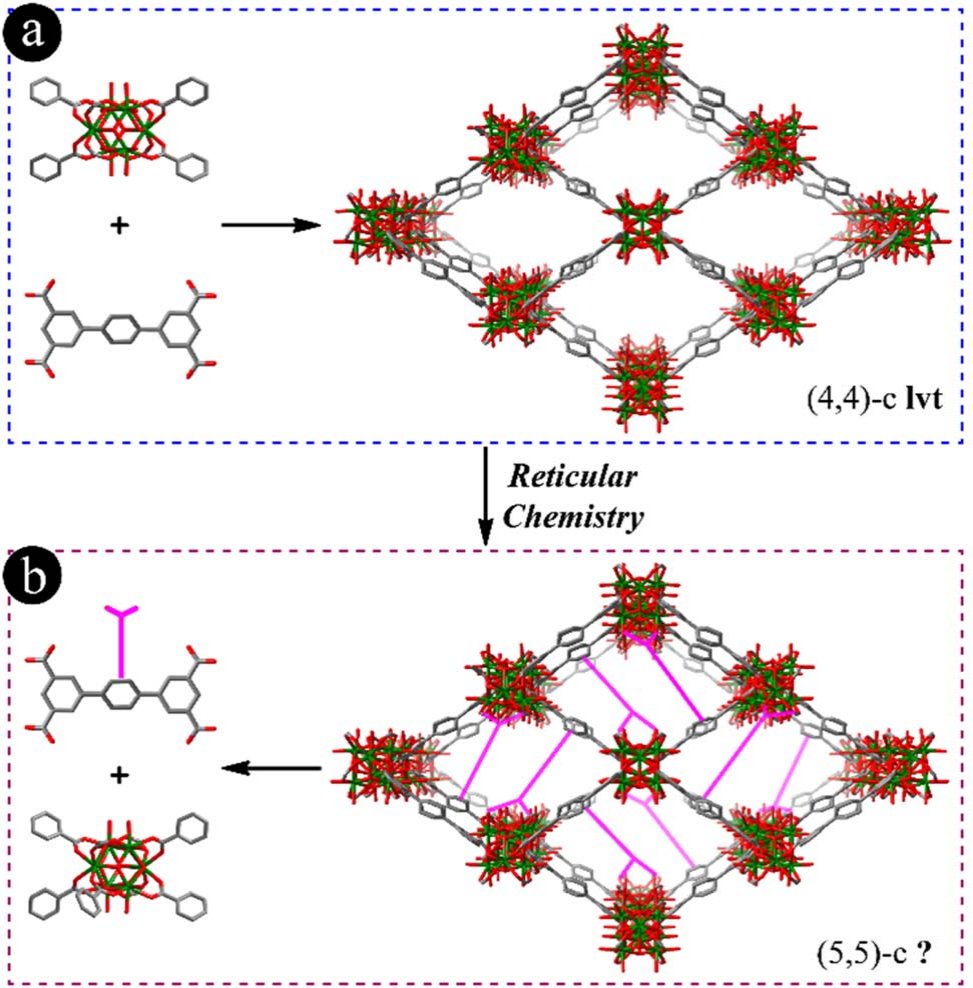
(a) Molecular structure of 4-connected [1,1′:4′,1′′]-terphenyl-3,3′′,5,5′′-tetracarboxylic acid (bottom left), 4-connected Zr_6_ cluster (top left) and single crystal structure of the corresponding MOF (lvt net, right); (b) the utilization of reticular chemistry to construct Zr-MOFs with 5-connected Zr_6_ clusters (bottom left) and the anticipated 5-connected linker structure (top left).

As reported and shown in Fig. 1a and S1,† Zr-MOFs with 4-connected Zr_6_ clusters have been constructed, such as NU-1400 with an lvt underlying net using [1,1′:4′,1′′]-terphenyl-3,3′′,5,5′′-tetracarboxylic acid (H_4_TPTC) or similar linkers.^19,26^ In these Zr-MOFs, each linker is coordinated with four Zr_6_ clusters and each Zr_6_ cluster is connected with four linkers, leading to the formation of rhombic channels along the *a* axis. From the view of reticular chemistry, TPTCs are packed between two rows of 4-connected Zr_6_ clusters in NU-1400 viewed from the *c* axis (Fig. S1c†). It is thus highly possible to add another carboxylate group on the central benzene ring of TPTC to coordinate with the neighboring Zr_6_ cluster and block the rhombic channels as depicted in Fig. 1b, in which the 5-connected Zr_6_ cluster and 5-connected pentacarboxylic acid will be generated.

## Results and discussion

**Fig. 2:**
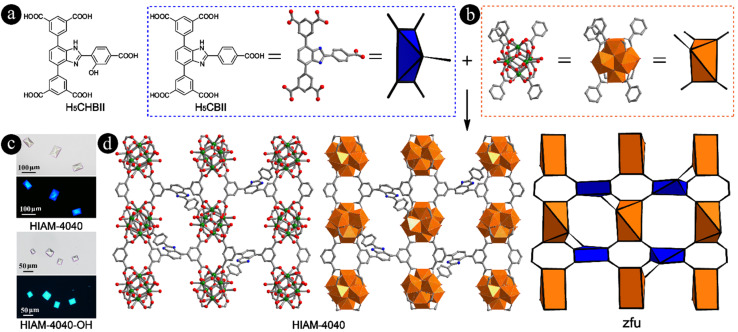
(a) Schematic representation of the molecular structures of H_5_CHBII and H_5_CBII; (b) 5-connected Zr_6_ cluster; (c) single crystal images of HIAM-4040 and HIAM-4040-OH under daylight (top) and 365 nm excitation (bottom); (d) the single-crystal structure of HIAM-4040 (color scheme: C, gray; O, red; Zr, green; N, blue).

Bearing the aforementioned consideration in mind, two pentacarboxylic acids, 5,5′-(2-(5-carboxythiophen-2-yl)-1*H*-benzo[*d*]imidazole-4,7-diyl)diisophthalic acid (H_5_CTBII) (Fig. S2†) and 5,5′-(2-(4-carboxyphenyl)-1*H*-benzo[*d*]imidazole-4,7-diyl)diisophthalic acid (H_5_CBII) (Fig. 2a), were designed and synthesized *via* Suzuki–Miyaura coupling followed by the saponification reaction (detailed procedures in the ESI, Fig. S3–S8†) to prove our hypothesis. We then attempted to synthesize Zr-MOFs using these two linkers. For H_5_CTBII, no crystals were obtained in spite of many attempts using various organic solvents and acid modulators. However, for H_5_CBII, single crystals with large size and uniform shape can be easily prepared. The typical synthesis procedure is as follows (Fig. 2a and b): a 5 mL vial containing H_5_CBII (0.018 mmol, 10.2 mg), ZrCl_4_ (0.10 mmol, 23.3 mg), 0.8 mL formic acid and 3 mL DMF was placed in a preheated oven at 120 °C for 3 days. Colorless single crystals of HIAM-4040 were obtained with bright blue emission under 365 nm excitation (Fig. 2c and S9†) (HIAM = Hoffmann Institute of Advanced Materials; 40 = zirconium).

The single crystal X-ray diffraction (sc-XRD) analysis at 193 K indicated that HIAM-4040 crystallizes in an orthorhombic crystal system with a *P*2_1_2_1_2_1_ space group (Table S1†). As expected, each Zr_6_O_8_ cluster in HIAM-4040 is coordinated by five fully deprotonated CBII linkers, three formate groups and four terminal H_2_O/OH^−^ groups (Fig. 2d, S10 and S11†), which was confirmed by the ^1^H NMR spectrum of digested HIAM-4040 (Fig. S12†). Each CBII is connected to five Zr_6_O_8_ clusters. As a result, HIAM-4040 possesses a rarely reported (5,5)-c zfu topology with the overall formula of Zr_6_O_4_(OH)_8_(H_2_O)_4_(HCOO)_3_(CBII). To the best of our knowledge, HIAM-4040 is the first example of Zr-MOFs constructed using a pentacarboxylate-type linker with 5-connected organic linkers and 5-connected Zr_6_ clusters, which is different from the reported 5-connected Zr_6_ cluster in NU-500, where one monodentate carboxylate ligand exists.^50,51^ A close structure analysis demonstrates that, similar to that in NU-1400, CBIIs are also packed between two rows of Zr_6_ clusters as viewed along the *a* axis, where additional carboxylate groups are connected to the left/below or the right/above the Zr_6_ cluster (Fig. S10†). As a result, the rhombic channels as observed in NU-1400 along the *a* axis are separated into two triangular-shaped channels and one rhombic-shaped channel along the *b* axis (Fig. S10,† middle). After removing the 4-(1*H*-imidazole-2-yl)benzoic acid group from the single crystal structure of HIAM-4040, the residual structure is almost the same as that of NU-1400 (Fig. S1 and S13†). The length of the added moiety containing carboxyl group in H_5_CBII is about 8.33 Å, while the distances between the central benzene ring of the organic linker and the neighboring Zr_6_ cluster are around 8.10 to 12.67 Å in NU-1400 under various conditions due to its flexibility. Therefore, NU-1400 can accommodate another carboxyl-contained moiety to generate the (5,5)-c underlying net. These results are consistent with our hypothesis that the 5-connected Zr_6_ clusters can be generated using pentacarboxylic acids based on reticular chemistry guided structural analysis of (4,4)-c lvt type Zr-MOFs, which further confirms that reticular chemistry is a powerful strategy for designing and discovering MOFs with novel structures.

To further investigate the tunability of linkers and their induced structure and property diversity, two other pentacarboxylic acids, 5,5′-(2-(4-carboxy-2-hydroxyphenyl)-1*H*-benzo[*d*]imidazole-4,7-diyl)diisophthalic acid (H_5_CHBII) (Fig. 2a) and 5,5′-(2-(4′-carboxybiphenyl-4-yl)-1*H*-benzo[*d*]imidazole-4,7-diyl)diisophthalic acid (H_5_CYBII) (Fig. S2†), were designed and synthesized *via* a similar method to H_5_CBII (detailed procedures in the ESI, Fig. S14–S19†). H_5_CHBII is the hydroxyl-functionalized H_5_CBII used to introduce excited-state intramolecular proton transfer (ESIPT). H_5_CYBII is the extension of H_5_CBII by incorporating an additional phenyl ring to the central benzene ring to investigate the tolerance of linker length for constructing (5,5)-c zfu type Zr-MOFs. As a result, light yellow single crystals (HIAM-4040-OH) were formed using H_5_CHBII as the organic linker, which shows bright cyan emission (Fig. 2c) with the overall formula of Zr_6_O_4_(OH)_8_(H_2_O)_4_(HCOO)_3_(CHBII) (Fig. S20†). However, for H_5_CYBII, no crystals were obtained, which might be ascribed to the fact that the length of the added moiety is too large to be accommodated in the structure of NU-1400 to generate the (5,5)-c net. These results indicate that the formation of (5,5)-c zfu type Zr-MOFs has stringent requirements on the geometry and length of organic linkers. The sc-XRD analysis at 193 K revealed that HIAM-4040-OH also crystallizes in an orthorhombic crystal system but with a different space group, *Pnma* (Table S2†).

**Fig. 3:**
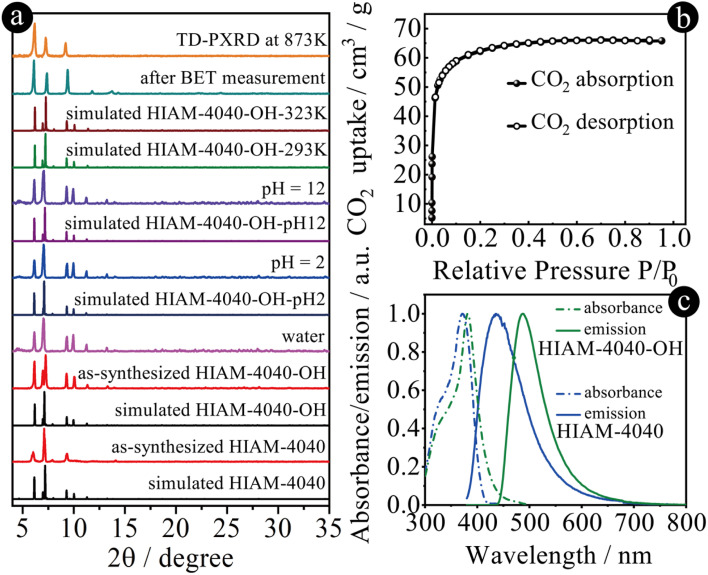
(a) Simulated and experimental PXRD patterns of HIAM-4040, HIAM-4040-OH and HIAM-4040-OH after treatment under various conditions; (b) CO_2_ adsorption–desorption isotherms at 195 K for HIAM-4040-OH; (c) solid-state emission spectra (solid lines) and UV-vis absorption spectra (dash lines) of HIAM-4040 (blue lines) and HIAM-4040-OH (green lines).

The phase purity of HIAM-4040 and HIAM-4040-OH was confirmed by the matched powder X-ray diffraction (PXRD) patterns between the simulated (193 K) and experimental ones (298 K) (Fig. 3a), which also reveal the isoreticular nature of HIAM-4040 and HIAM-4040-OH. A small peak shift was observed in the PXRD pattern of the activated HIAM-4040-OH sample after porosity analysis, indicative of its structural flexibility. The chemical and thermal stability of these two MOFs were also tested. As depicted in Fig. 3a and S21,† the intact PXRD patterns demonstrate that the long-range orders of HIAM-4040 and HIAM-4040-OH were well retained after treatment under various aqueous conditions, including soaking in water, in pH = 2 and 12 solutions at room temperature for 24 hours, respectively. Due to the relatively lower yield of HIAM-4040 compared with that of HIAM-4040-OH, the *in situ* temperature-dependent PXRD (TD-PXRD) and porosity measurements were conducted using HIAM-4040-OH. As depicted in Fig. 3a and S22,† the crystallinity of HIAM-4040-OH remained unchanged even upon heating to 873 K, which is consistent with the thermogravimetric analysis (Fig. S23†). These results demonstrate that HIAM-4040 and HIAM-4040-OH have excellent chemical and thermal stability. To further confirm the framework stability, we obtained single crystal structures of HIAM-4040-OH after various treatments, namely HIAM-4040-OH-293K, HIAM-4040-OH-323K, HIAM-4040-OH-EtOH (after solvent exchange using EtOH), HIAM-4040-OH-pH2, and HIAM-4040-OH-pH12. As summarized in Tables S1 to S8,† the sc-XRD analyses not only confirm structural stability of HIAM-4040-OH upon heating and exposure to harsh chemical environments, but also indicate temperature- and solvent-dependent structural flexibility (Fig. 3a, S22 and S24†), which is similar to NU-1400.^26^

We attempted to analyze the permanent porosity of HIAM-4040-OH by N_2_ and Ar sorption experiments; however negligible gas uptake was observed. We then carried out CO_2_ sorption measurement at 195 K due to its smaller kinetic diameter compared with N_2_ and Ar, which exhibited a type I adsorption profile (Fig. 3b). The corresponding BET surface area and total pore volume were estimated to be 308.2 m^2^ g^−1^ and 0.14 cm^3^ g^−1^, respectively, smaller than those of NU-1400, due to the introduction of 4-(1*H*-imidazole-2-yl)benzoic acid. The pore size distribution analysis indicated two types of pores with estimated sizes of 4.5 and 6.6 Å (Fig. S25†). The experimental BET surface area and pore volume are much smaller than the calculated values of 2626.0 m^2^ g^−1^ and 0.56 cm^3^ g^−1^, which demonstrates that only a small amount of porosity was accessible for HIAM-4040-OH.

The solid-state UV-vis absorption experiments revealed that the adsorption maxima are at 372 nm and 382 nm for HIAM-4040 and HIAM-4040-OH, respectively (Fig. 3c). The maximum emission peaks center at 436 nm and 487 nm for HIAM-4040 and HIAM-4040-OH, respectively. It should be noticed that a Stokes shift of 105 nm was observed for HIAM-4040-OH, which is 41 nm larger than that of HIAM-4040 (64 nm). These results indicate that the introduction of a hydroxyl group triggers ESIPT and leads to red-shift emission from the keto form of CHBII under excitation (Fig. S26†),^52^ which is the same as we have observed in the previous work.^47^ The photoluminescence quantum yields are 24.7% and 26.3% for HIAM-4040 and HIAM-4040-OH under 365 nm excitation, respectively.

**Fig. 4:**
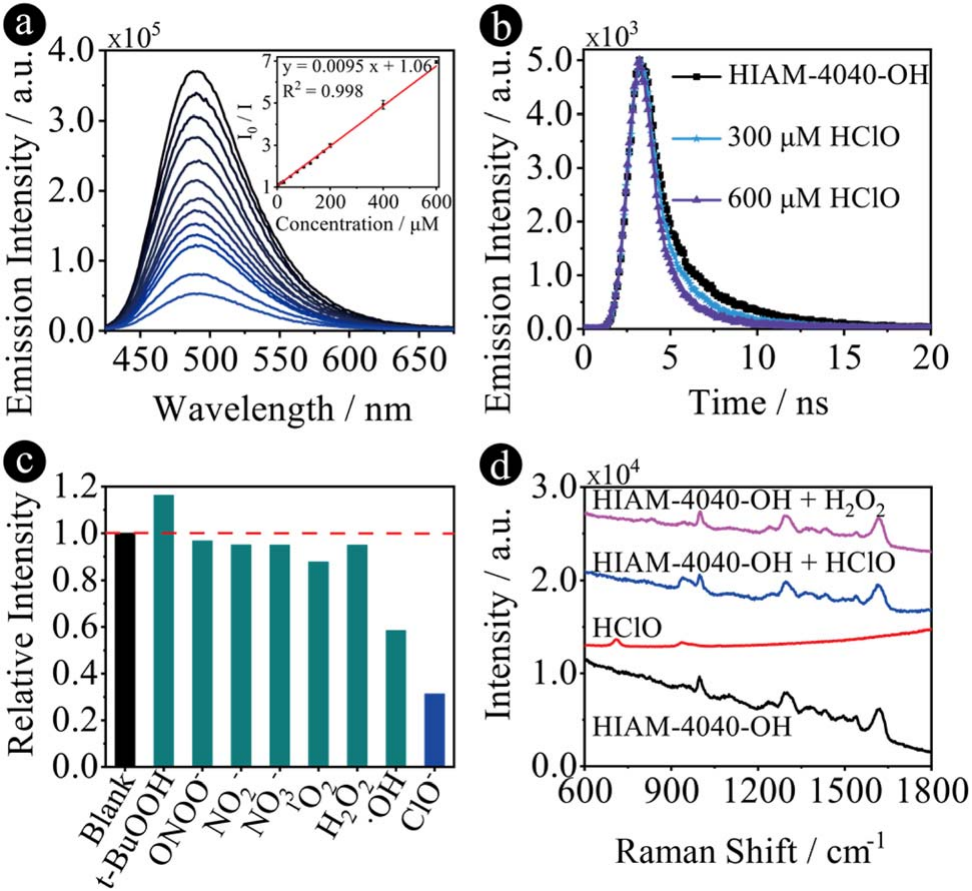
(a) The concentration-dependent emission quenching of HIAM-4040-OH with HClO concentrations of 5, 15, 25, 50, 75, 100, 125, 150, 175, 200, 400 and 600 μM, and the corresponding Stern–Volmer plot; (b) the fluorescence lifetime decay profiles of HIAM-4040-OH before and after addition of HClO; (c) the selectivity test of HIAM-4040-OH with different kinds of ROS and RNS; (d) the Raman spectra of HIAM-4040-OH before and after treatment using HClO and H_2_O_2_.

Due to strong emission and excellent chemical stability, especially in aqueous solution, HIAM-4040-OH was used to detect hypochlorous acid (HClO), one of the reactive oxygen species (ROS). HClO and other ROS and reactive nitrogen species (RNS) play an essential role in biology,^53–56^ where the production of excess HClO is related to various diseases. Therefore, it is of great interest to monitor the concentration of HClO under aqueous conditions. The HClO concentration-dependent emission spectra were thus measured by gradual addition of HClO into the aqueous suspension of HIAM-4040-OH. As shown in Fig. 4a, the emission at 490 nm of HIAM-4040-OH gradually decreased with an increasing concentration of HClO. This result is consistent with the decreased emission lifetime of HIAM-4040-OH after addition of HClO (Fig. 4b), which decreased from 2.00 ns to 1.58 ns and 1.24 ns when 300 and 600 μM HClO was added. The emission intensity of HIAM-4040-OH showed a linear correlation coefficient of 0.998 toward the concentration of HClO in the range of 0 to 0.6 mM with a calculated detection limitation of 1.57 μM, which is comparable with those of the reported MOF-based materials for HClO detection (Table S9†). A similar emission quenching behavior was also observed for HIAM-4040 after adding HClO (Fig. S27†). HIAM-4040-OH also exhibited high selectivity towards HClO compared with other RNS and ROS, including t-BuOOH, ONOO^−^, NO_2_^−^, NO_3_^−^, ^1^O_2_, H_2_O_2_ and ·OH (Fig. 4c). Moreover, the well matched PXRD patterns of the simulated HIAM-4040-OH and as-synthesized HIAM-4040-OH, after grinding and detection of HClO indicate the excellent stability of HIAM-4040-OH (Fig. S28†). These results demonstrate that HIAM-4040-OH can be used for highly sensitive and selective detection of HClO in aqueous solutions.

To understand the turn-off sensing mechanism of HIAM-4040-OH toward HClO, several control experiments were conducted. (i) The similar emission quenching behavior of HIAM-4040 and HIAM-4040-OH upon the addition of HClO indicates that the OH group on the linker skeleton of HIAM-4040-OH is not the reaction site for the turn-off mechanism (Fig. S27†). (ii) Almost no change was observed for the emission of HIAM-4040-OH when HCl was added, indicating that the emission quenching is not from the acidic nature of HClO (Fig. S29†). (iii) A slight emission decrease was recorded when HClO was added into a benzothiadiazole-based luminescent MOF solution, HIAM-4011,^57^ which demonstrates that the N atom on the benzothiadiazole is silent toward HClO (Fig. S30†). (iv) The UV-vis absorption range of HClO is less than 350 nm, ruling out the energy transfer induced emission quenching between HIAM-4040-OH and HClO (Fig. S31†). According to the aforementioned experiments, we conclude that the reason for the turn-off emission is the reaction between HClO and the NH group on the imidazole ring. As a strong oxidizing agent, the NH group will be oxidized to N after adding HClO to HIAM-4040-OH solution, where the electrons will rearrange to generate a carbon radical, which will quench the emission of HIAM-4040-OH. To confirm our hypothesis, the Raman spectra of HIAM-4040-OH before and after adding HClO were measured. As shown in Fig. 4d, the typical Raman peaks at 998, 1295 and 1617 cm^−1^ were attributed to the trigonal bending, C–N and C=C stretching vibrations of linkers in HIAM-4040-OH. After addition of HClO, a new Raman peak originating from C–N–C appeared at 940 cm^−1^ and was attributed to the oxidation of N–H on the imidazole ring, while no peak at 940 cm^−1^ was observed when H_2_O_2_ was added, which further confirms that only HClO has this specific interaction with HIAM-4040-OH.

## Conclusion

In conclusion, we report the successful application of reticular chemistry to construct pentacarboxylate-based Zr-MOFs, HIAM-4040 and HIAM-4040-OH, inspired by (4,4)-c lvt type Zr-MOFs. To the best of our knowledge, this is the first Zr-pentacarboxylate framework and the first study disclosing a 5-connected organic linker and 5-connected Zr_6_ node. By introducing a hydroxyl group into the linker skeleton of HIAM-4040, an isoreticular structure, HIAM-4040-OH, is generated with a larger Stokes shift *via* ESIPT. Due to its excellent chemical and thermal stability, HIAM-4040-OH was utilized for HClO detection in an aqueous solution with high sensitivity and selectivity. This work points to a new avenue for rationally designing and constructing Zr-MOFs with unique structures guided by reticular chemistry.

## Data availability

All the data can be found in the main article and ESI.†

## Author contributions

X. Y. L. conceived the idea and designed the experiment; T. Peng and C.-Q. Han worked on the synthesis and the characterization of all materials. H.-L. Xia and K. Zhou obtained and analyzed the single crystal structures. J. Zhang, J. Si, L. Wang and L.-L. Qu performed the data analysis of HClO detection. J. Miao, F.-A. Guo and H. Wang obtained the adsorption–desorption isotherms. X.-Y. L., J. Li and G. Xu wrote the paper with help from all authors. T. Peng and C.-Q. Han contributed equally to this work.

## Conflicts of interest

The authors declare no competing financial interests.

## Supplementary Material

SC-015-D3SC05410A-s001

SC-015-D3SC05410A-s002
