# Supplementary material for: Reticular chemistry guided precise construction of zirconium-pentacarboxylate frameworks with 5-connected Zr6 clusters
Source: Chem Sci. 2024 Jan 23;15(9):3174–81. doi: 10.1039/d3sc05410a (PMC10901486; doi:10.1039/d3sc05410a)
Supplement: SC-015-D3SC05410A-s001 [file SC-015-D3SC05410A-s001.pdf]

Electronic Supplementary Material (ESI) for Chemical Science.
This journal is © The Royal Society of Chemistry 2024

Supporting Information

Reticular Chemistry Guided Precise Construction of Zirconium-Pentacarboxylate Frameworks with 5-Connected Zr_6 Clusters

Tianyou Peng,[†,‡,+] Chao-Qin Han,[†,+] Hai-Lun Xia,[†] Kang Zhou,[†] Jian Zhang,[†] Jincheng Si,[§,†] Lei Wang,[†] Jiafeng Miao,[†] Fu-An Guo,[†] Hao Wang,[†] Lu-Lu Qu,[§] Guozhong Xu,[*,‡] Jing Li,[*,¶,†] and Xiao-Yuan Liu [*,†]

[†] Hoffmann Institute of Advanced Materials, Shenzhen Polytechnic University, 7098 Liuxian Blvd, Nanshan District, Shenzhen, 518055, P. R. China

[‡] College of Chemical Engineering, University of Science and Technology Liaoning, Anshan 114051, P. R. China

[§] School of Chemistry & Materials Science, Jiangsu Normal University, Xuzhou, 221116, P. R. China

[¶] Department of Chemistry and Chemical Biology, Rutgers University, 123 Bevier Road, Piscataway, New Jersey 08854, United States

* To whom correspondence should be addressed: gz_xu@ustl.edu.cn, jingli@rutgers.edu and

liuxiaoyuan1989@szpu.edu.cn

Chemicals

3,6-dibromobenzene-1,2-diamine (97%), (3,5-bis(methoxycarbonyl)phenyl)boronic acid (97%), methyl 4-formyl-3-hydroxybenzoate (95%), dimethyl 5-(4,4,5,5-tetramethyl-1,3,2-dioxaborolan-2-yl)isophthalate (98%), methyl 5-formylthiophene-2-carboxylate (98%) and methyl 4'-formylbiphenyl-4-carboxylate (98%) were purchased from Shanghai Bidepharm Co., Ltd. Zirconium (IV) chloride ($ZrCl_4$) (99.5%), N,N-dimethylformamide (DMF) (99.5%) and N,N-diethylformamide (DEF) (99%) were purchased from Shanghai Macklin Biochemical Technology Co., Ltd. Formic acid (98%) were purchased from Shanghai Aladdin Biochemical Technology Co., Ltd. Methyl 4-(4,7-dibromo-1H-benzo[d]imidazol-2-yl)benzoate was synthesized according to the previous work.[1] All the other chemicals were obtained from the chemical supplies and used without further purification.

Characterization

Nuclear magnetic resonance (NMR) data was recorded using 400 MHz JEOL JNM-ECZ400S. Single crystal X-ray diffraction data were collected on a Bruker D8 Venture diffractometer. Powder X-ray diffraction (PXRD) patterns were measured using Bruker D8 Advance X-ray diffractometer with Cu Kα radiation. Photoluminescent and UV-vis spectra were measured on FLS1000 spectrofluorometer (Edinburgh Instruments) and Shimadzu UV-3600 spectrophotometer, respectively. Raman spectra were acquired using the confocal Raman spectrometer XploRA (HORIBA, Japan) with the 785 nm excitation laser. Elemental analyses (EA) were collected using the Elementar Vario EL cube.

Synthesis of methyl 5-(4,7-dibromo-1H-benzo[d]imidazol-2-yl)thiophene-2-carboxylate

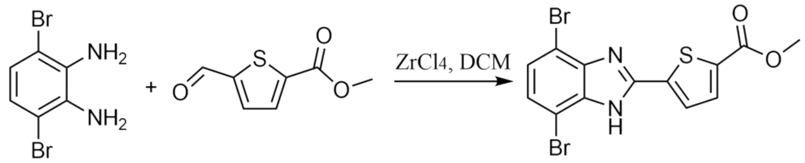

3,6-dibromobenzene-1,2-diamine (10.0 mmol, 2.66 g), methyl 5-formylthiophene-2-carboxylate (11.0 mmol, 1.87 g), $ZrCl_4$ (0.50 mmol, 0.12 g) were added into a flask containing 30 mL DCM. The mixture was stirred at room temperature for 48 h. A light-yellow precipitate was gradually formed with increased reaction time. The precipitate was filtered and washed using DCM to give methyl 5-(4,7-dibromo-1H-benzo[d]imidazol-2-yl)thiophene-2-carboxylate as a light-yellow solid (3.89 g, 94.2%). ^{1}H NMR (400 MHz, DMSO-d_6) δ ppm 8.14 (1H), 7.87 (1H), 7.38 (2H), 3.84 (3H).

Synthesis of tetramethyl 5,5'-(2-(5-(methoxycarbonyl)thiophen-2-yl)-1H-benzo[d] imidazole-4,7-diyl)diisophthalate

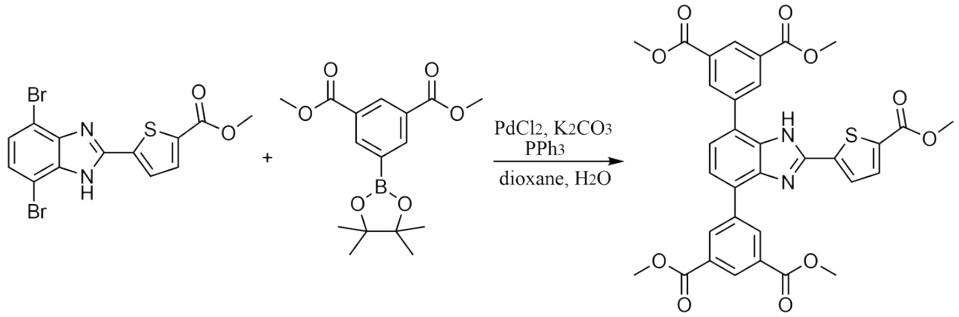

Methyl 5-(4,7-dibromo-1H-benzo[d]imidazol-2-yl)thiophene-2-carboxylate (5.0 mmol, 2.07 g), dimethyl 5-(4,4,5,5-tetramethyl-1,3,2-dioxaborolan-2-yl)isophthalate (11.0 mmol, 3.52 g), $PdCl_2$ (0.4 mmol, 70.0 mg), PPh_3 (0.8 mmol, 0.21 g) and K_2CO_3 (16.0 mmol, 2.20 g) were added into one 250 mL round-bottle flask containing 120 mL dioxane and 30mL water. The mixture was degassed four times and stirred at 105°C for 16 hours. The precipitates were filtered to offer tetramethyl 5,5'-(2-(5-(methoxycarbonyl)thiophen-2-yl)-1H-benzo[d]imidazole-4,7-diyl)diisophthalate as a light yellow solid (2.65 g, 82.6%), which was directly used for next step without further purification. ^{1}H NMR (400 MHz, DMSO-d_6) δ ppm 8.89 (4H), 8.41 (2H), 7.86 (1H), 7.76 (1H), 7.44 (2H), 3.90 (12H), 3.82 (3H).

Synthesis of 5,5'-(2-(5-carboxythiophen-2-yl)-1H-benzo[d]imidazole-4,7-diyl) diisophthalic acid (H_5CTBII)

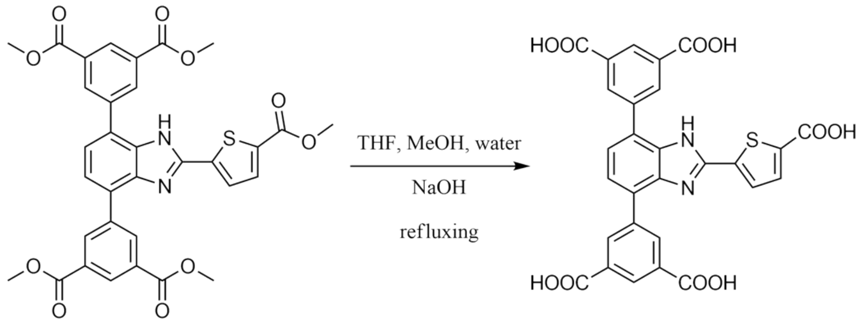

Tetramethyl 5,5'-(2-(5-(methoxycarbonyl)thiophen-2-yl)-1H-benzo[d] imidazole- 4,7-diyl)diisophthalate (4.0 mmol, 2.57 g) was added to a solution containing 20 mL CH_3OH, 50 mL THF and 50 mL water with 3.5 g NaOH. The mixture was heated to reflux at 85 °C overnight. After cooling down to room temperature, the organic solvent was removed under reduced pressure. Then aqueous solution was neutralized using 2M HCl to obtain the precipitate, which was filtered to offer the final product 5,5'-(2-(5-carboxythiophen-2-yl)-1H-benzo[d]imidazole-4,7-diyl)diisophthalic acid (H_5CTBII) as a yellow solid (2.18 g, 95.3%). ^1H NMR (400 MHz, DMSO-d_6) δ ppm 8.98 (2H), 8.51 (2H), 8.38 (2H), 8.04 (1H), 7.72 (1H), 7.67 (1H), 7.44 (1H). ^3C NMR (101 MHz, DMSO-d_6) δ ppm 167.15, 163.24, 148.09, 139.42, 138.84, 136.77, 134.49, 133.93, 132.35, 129.58, 128.95.

Synthesis of tetramethyl 5,5'-(2-(4-(methoxycarbonyl)phenyl)-1H-benzo [d]imidazole-4,7-diyl)diisophthalate

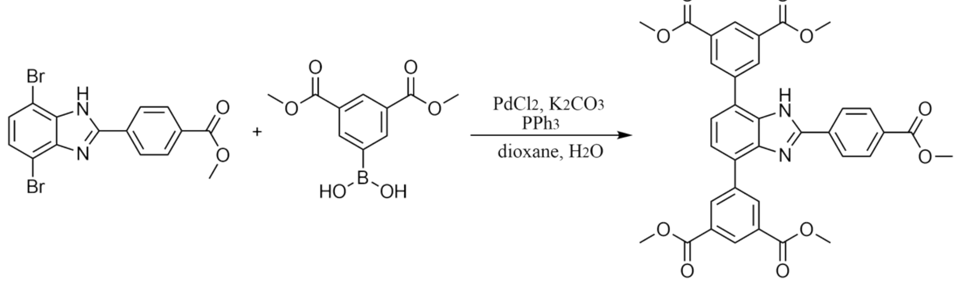

Methyl 4-(4,7-dibromo-1H-benzo[d]imidazol-2-yl)benzoate (5.0 mmol, 2.04 g), (3,5-bis(methoxycarbonyl)phenyl)boronic acid (11.0 mmol, 2.62 g), $PdCl_2$ (0.4 mmol, 70.0 mg), PPh_3 (0.8 mmol, 0.21 g) and K_2CO_3 (16.0 mmol, 2.20 g) were added into one 250 mL round-bottle flask containing 120 mL dioxane and 30mL water. The mixture was degassed four times and stirred at 105°C for 24 hours. After cooling down to room temperature, the precipitates were filtered to offer tetramethyl 5,5'-(2-(4-(methoxycarbonyl)phenyl)-1H-benzo[d]imidazole-4,7-diyl)diisophthalate as a white solid (2.14 g, 67.3%), which was directly used for next step without further purification.

Synthesis of 5,5'-(2-(4-carboxyphenyl)-1H-benzo[d]imidazole-4,7-diyl)diisophthalic acid (H_5CBII)

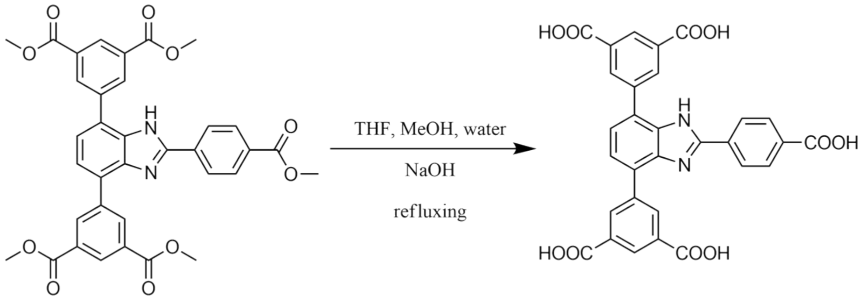

Tetramethyl 5,5'-(2-(4-(methoxycarbonyl)phenyl)-1H-benzo[d]imidazole-4,7-diyl) diisophthalate (3.0 mmol, 1.91 g) was added to a solution containing 20 mL CH_3OH, 50 mL THF and 50 mL water with 3.0 g NaOH. The mixture was heated to reflux at 85 °C overnight. After cooling down to room temperature, the organic solvent was removed under reduced pressure. Then aqueous solution was neutralized using 2M HCl to obtain the precipitate, which was filtered to offer the final product 5,5'-(2-(4-carboxyphenyl)-1H-benzo[d]imidazole-4,7-diyl)diisophthalic acid (H_5CBII) as a light-green solid (1.61 g, 94.8%). ^{1}H NMR (400 MHz, DMSO-d_6) δ ppm 8.69 (4H), 8.52 (2H), 8.38 (2H), 8.07 (2H), 7.56 (2H).

^{13}C NMR (101 MHz, DMSO-d_6) δ ppm 167.38, 167.16, 152.42, 138.92, 134.05, 133.73, 132.53, 132.05, 130.11, 129.55, 127.73.

Synthesis of methyl 4-(4,7-dibromo-1H-benzo[d]imidazol-2-yl)-3-hydroxybenzoate

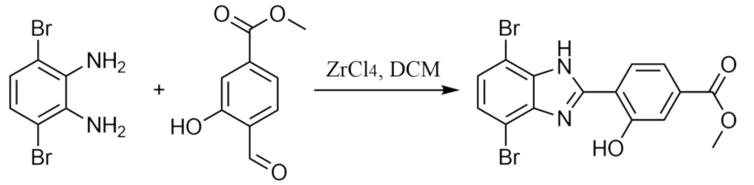

3,6-dibromobenzene-1,2-diamine (15.0 mmol, 3.96 g), methyl 4-formyl-3-hydroxybenzoate (16.0 mmol, 2.88 g), $ZrCl_4$ (0.75 mmol, 0.18 g) were added into a flask containing 50 mL DCM. The mixture was stirred at room temperature for 48 h. A yellow precipitate was gradually formed with increased reaction time. The precipitate was filtered and washed using DCM to give methyl 4-(4,7-dibromo-1H-benzo[d]imidazol-2-yl)-3-hydroxybenzoate as a light-yellow solid (5.96 g, 92.8%). ^{1}H NMR (400 MHz, DMSO-d_6) δ ppm 8.47 (1H), 7.54 (1H), 7.51 (1H), 7.44 (2H), 3.84 (3H).

Synthesis of tetramethyl 5,5'-(2-(2-hydroxy-4-(methoxycarbonyl)phenyl)-1H-benzo [d]imidazole-4,7-diyl)diisopht-halate

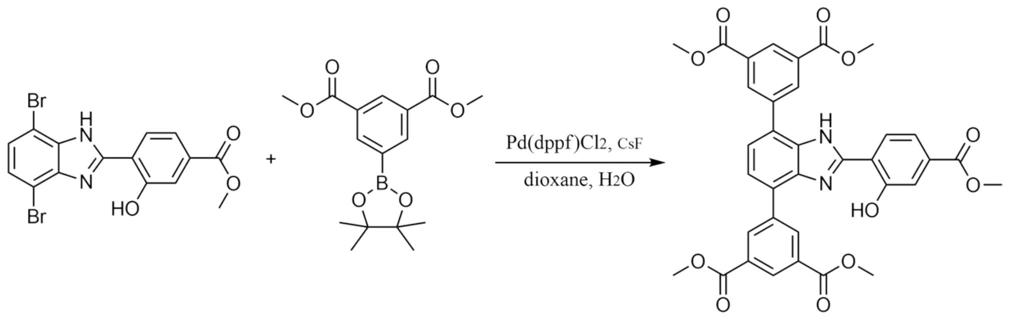

Methyl 4-(4,7-dibromo-1H-benzo[d]imidazol-2-yl)-3-hydroxybenzoate (5.0 mmol, 2.13 g), dimethyl 5-(4,4,5,5-tetramethyl-1,3,2-dioxaborolan-2-yl)isophthalate (11.0 mmol, 3.52 g), Pd(dppf)Cl_2 (0.5 mmol, 0.36 g), and CsF (30.0 mmol, 4.55 g) were added into one 250 mL round-bottle flask containing 80 mL dioxane and 40mL water. The mixture was degassed four times and stirred at 105°C for 16 hours. The reaction mixture was filtrated to offer

tetramethyl-5,5'-(2-(2-hydroxy-4-(methoxycarbonyl)phenyl)-1H-benzo[d]imidazole-4,7-diyl)diisophthalate as a light-yellow solid (2.67 g, 82.2%), which was directly used for next step without further purification.

Synthesis of 5,5'-(2-(4-carboxy-2-hydroxyphenyl)-1H-benzo[d]imidazole-4,7-diyl) diisophthalic acid (H_5CHBII)

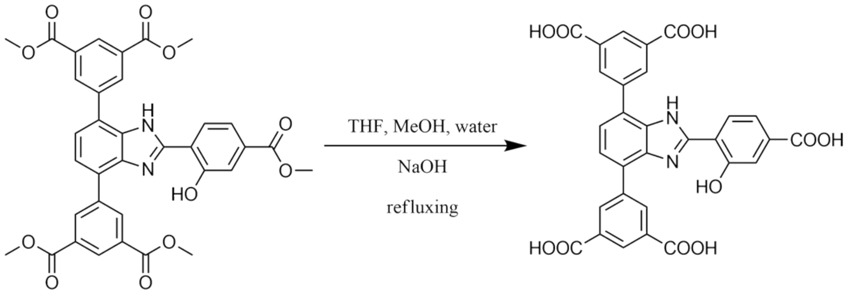

Tetramethyl-5,5'-(2-(2-hydroxy-4-(methoxycarbonyl)phenyl)-1H-benzo[d]imidazole-4,7-diyl)diisophthalate (4.0 mmol, 2.61 g) was added to a solution containing 15 mL CH_3OH, 30 mL THF and 50 mL water with 3.5 g NaOH. The mixture was heated to reflux at 85 °C overnight. After cooling down to room temperature, the organic solvent was removed under reduced pressure. Then aqueous solution was neutralized using 2M HCl to obtain the precipitate, which was filtered to offer the final product 5,5'-(2-(4-carboxy-2-hydroxyphenyl)-1H-benzo[d]imidazole-4,7-diyl)diisophthalic acid (H_5CHBII) as a light-yellow solid (2.11 g, 93.2%). ^{1}H NMR (400 MHz, DMSO-d_6) δ ppm 8.58 (4H), 8.54 (2H), 8.38 (1H), 7.60 (2H), 7.50 (2H). ^{13}C NMR (101 MHz, DMSO-d_6) δ ppm 167.19, 167.11, 158.27, 152.68, 138.63, 134.27, 133.85, 132.48, 129.71, 128.19, 120.09, 118.27, 116.49.

Synthesis of methyl 4'-(4,7-dibromo-1H-benzo[d]imidazol-2-yl)biphenyl-4-carboxylate

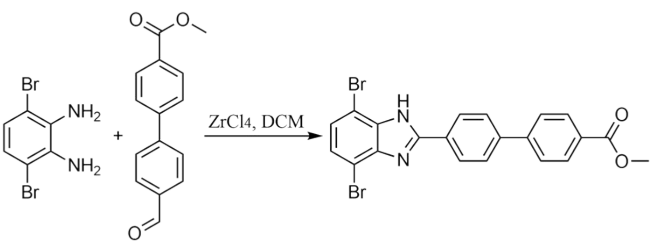

3,6-dibromobenzene-1,2-diamine (15.0 mmol, 3.96 g), methyl 4'-formylbiphenyl-4-carboxylate (16.0 mmol, 3.84 g), $ZrCl_4$ (0.75 mmol, 0.18 g) were added into a flask containing 50 mL DCM. The mixture was stirred at room temperature for 48 h. A light-yellow precipitate was gradually formed with increased reaction time. The precipitate was filtered and washed using DCM to give methyl 4'-(4,7-dibromo-1H-benzo[d]imidazol-2-yl)biphenyl-4-carboxylate as a light-yellow solid (6.28 g, 86.7%). ^{1}H NMR (400 MHz, DMSO-d_6) δ ppm 8.43 (2H), 8.05 (2H), 7.94 (4H), 7.37 (2H), 3.86 (3H).

Synthesis of tetramethyl 5,5'-(2-(4'-(methoxycarbonyl)biphenyl-4-yl)-1H-benzo [d]imidazole-4,7-diyl)diisophthalate

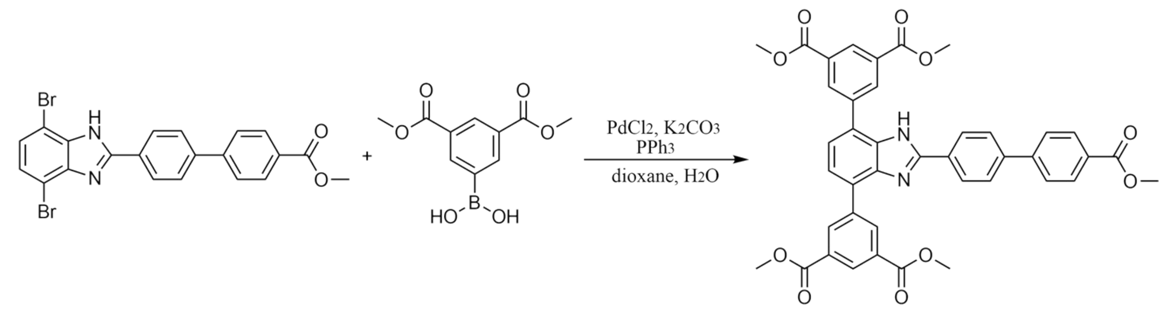

Methyl 4'-(4,7-dibromo-1H-benzo[d]imidazol-2-yl)biphenyl-4-carboxylate (5.0 mmol, 2.42 g), (3,5-bis(methoxycarbonyl)phenyl)boronic acid (11.0 mmol, 2.62 g), $PdCl_2$ (0.4 mmol, 70.0 mg), PPh_3 (0.8 mmol, 0.21 g) and K_2CO_3 (16.0 mmol, 2.20 g) were added into one 250 mL round-bottle flask containing 120 mL dioxane and 30mL water. The mixture was degassed four times and stirred at 105°C for 24 hours. After cooling down to room temperature, the precipitates were filtered to offer tetramethyl 5,5'-(2-(4'-(methoxycarbonyl)biphenyl-4-yl)-1H-benzo[d]imidazole-4,7-diyl)diisophthalate as a light-green solid (2.84 g, 79.8%), which was directly used for next step without further purification.

Synthesis of 5,5'-(2-(4'-carboxybiphenyl-4-yl)-1H-benzo[d]imidazole-4,7-diyl) diisophthalic acid (H_5CYBII)

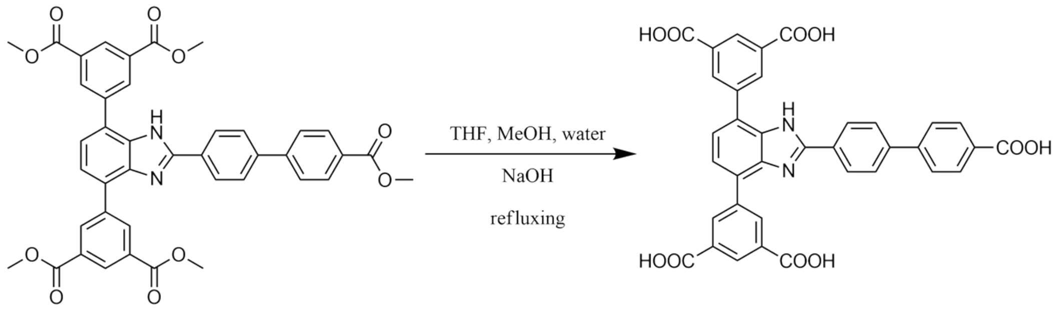

Tetramethyl 5,5'-(2-(4'-(methoxycarbonyl)biphenyl-4-yl)-1H-benzo[d] imidazole-4,7-diyl) diisophthalate (2.0 mmol, 1.43 g) was added to a solution containing 15 mL CH_3OH, 30 mL THF and 50 mL water with 2.0 g NaOH. The mixture was heated to reflux at 85 °C overnight. After cooling down to room temperature, the organic solvent was removed under reduced pressure. Then aqueous solution was neutralized using 2M HCl to obtain the precipitate, which was filtered to offer the final product 5,5'-(2-(4'-carboxybiphenyl-4-yl)-1H-benzo[d]imidazole-4,7-diyl)diisophthalic acid (H5CYBII) as a light-green solid (1.19 g, 92.7%). ^{1}H NMR (400 MHz, DMSO-d_6) δ ppm 8.71 (4H), 8.53 (2H), 8.39 (2H), 8.01 (2H), 7.93 (2H), 7.88 (2H), 7.55 (2H). ^{13}C NMR (101 MHz, DMSO-d_6) δ ppm 167.61, 167.21, 153.01, 143.85, 140.94, 139.05, 134.04, 132.29, 130.53, 129.84, 129.50, 128.48, 127.86, 127.43.

Synthesis of HIAM-4040 single crystals

$ZrCl_4$ (0.10 mmol, 23.3 mg), H_5CBII (0.018 mmol, 10.2 mg), formic acid (0.8 mL) and DMF (3.0 mL) were sequentially added into a 5 mL vial at room temperature. After sonicated for 5 minutes, the vial was directly put into a preheated oven at 120°C for 3 days. After cooling down to room temperature, the colorless single crystals were obtained, which was washed using DMF for several times to completely removing the suspended matters and until no

fluorescent single was detected from the supernatant (yield: about 10%).

Synthesis of HIAM-4040-OH single crystals

$ZrCl_4$ (0.13 mmol, 30.3 mg), H_5CHBII (0.018 mmol, 10.5 mg), formic acid (2.0 mL) and DEF (5.0 mL) were sequentially added into a 20 mL vial at room temperature. After sonicated for 5 minutes, the vial was directly put into a preheated oven at 120°C for 4 days. After cooling down to room temperature, the light-yellow single crystals were obtained, which was washed using DMF for several times until no fluorescent single was detected from the supernatant (yield: about 83%).

Single-crystal X-ray diffraction analyses

Single crystals of HIAM-4040, HIAM-4040-OH, HIAM-4040-OH-EtOH, HIAM-4040-OH-pH2, HIAM-4040-OH-pH12 were mounted on MicroMesh (MiTeGen) with paraton oil. The data were collected on a ' Bruker D8 VENTURE ' diffractometer equipped with gallium micro-focus metaljet X-ray sources (λ = 1.34139 Å for HIAM-4040), Mo micro-focus X-ray sources (λ = 0.71073 Å for HIAM-4040-OH, HIAM-4040-OH-EtOH, HIAM-4040-OH-pH2

and HIAM-4040-OH-pH12) or Cu micro-focus X-ray sources (λ = 1.54178 Å for HIAM-4040-OH at 293K and 323K). The crystals kept at the 193 K during data collection for HIAM-4040, HIAM-4040-OH, HIAM-4040-OH-EtOH, HIAM-4040-OH-pH2, HIAM-4040-OH-pH12. The single-crystal structures of HIAM-4040-OH were also obtained at 293 and 323 K. Using Olex2[2], the structures were solved with the ShelXT[3] structure solution program using Intrinsic Phasing and refined with the ShelXL[4] refinement package using Least Squares minimization. In all of structures, pentacarboxylate ligands are found to be disordered over two (H_5CBII) or four (H_5CHBII) part with occupancy of 0.5:0.5 (H_5CBII) or 0.25:0.25:0.25:0.25 (H_5CHBII). The restraints SIMU, SADI, DFIX, DANG, FLAT and ISOR were used to make the ADP values of those disordered atoms more reasonable. The hydrogen atoms on the aromatic rings were located at geometrically calculated positions and refined by riding. However, the hydrogen atoms for the coordinated molecules cannot be found from the residual electron density peaks and the attempt of theoretical addition was not done. The free solvent molecules are highly disordered in MOFs, and attempts to locate and refine the solvent peaks were unsuccessful. The diffused electron densities resulting from these solvent molecules were removed using the Solvent Mask routine of Olex2, structures were then refined again using the data generated. The refinement results are summarized in Table S1-S7. Crystallographic data for all of the crystal structures in CIF format have been deposited in the Cambridge Crystallographic Data Centre (CCDC) under deposition number CCDC-2274419 (HIAM-4040), 2290437 (HIAM-4040-OH-193K), 2290440 (HIAM-4040-OH-293K), 2290438 (HIAM-4040-OH-323K), 2290436 (HIAM-4040-OH-EtOH), 2290439 (HIAM-4040-OH-pH2), 2290441 (HIAM-4040-OH-pH12). The data can be obtained free of charge

via www.ccdc.cam.ac.uk/data_request/cif (or from the Cambridge Crystallographic Data Centre, 12 Union Road, Cambridge CB2 1EZ, U.K.)

Chemical stability measurement

The synthesized HIAM-4040-OH single crystals were washed with fresh DMF for 24 hours to remove unreacted organic linkers and inorganic matters. The DMF was removed and the crystals were immersed in pH = 2, pH = 7, pH = 12 aqueous solutions (3 mL) for 24 hours. The aqueous solution was then removed and the crystals were washed with DMF, which were used for single-crystal X-ray diffraction analyses or filtrated for further measurements.

Temperature-dependent PXRD (TD-PXRD) Analyses

In situ PXRD measurements were performed on a powder X-ray diffraction analyzer at a voltage of 50 kV, a current of 40 mA, a time of 0.15 s/step, a 2θ of 3 to 40 degree, a ramp rate of 10 °C/min, and a temperature range of 30 (303 K) to 600 °C (873 K).

CO_2 sorption isotherms

CO_2 sorption isotherms measurements were carried out using a Conta specific surface area and pore size analyzer. As-synthesized HIAM-4040-OH single crystals were washed using fresh DMF for 24 hours to remove unreacted organic linkers and inorganic species. Then was exchanged for 24 hours with ethanol. During the exchange process, the solvent was changed several times for complete replacement. The sample was then dried in a dynamic vacuum at 100 °C for 2 hours. The low pressure CO_2 adsorption isotherm was measured in a mixed bath of acetone and dry ice at 195 K. The specific surface areas were calculated using the Brunauer-Emmett-Teller model based on the CO_2 sorption data and Calculation of pore size distribution using HK model.

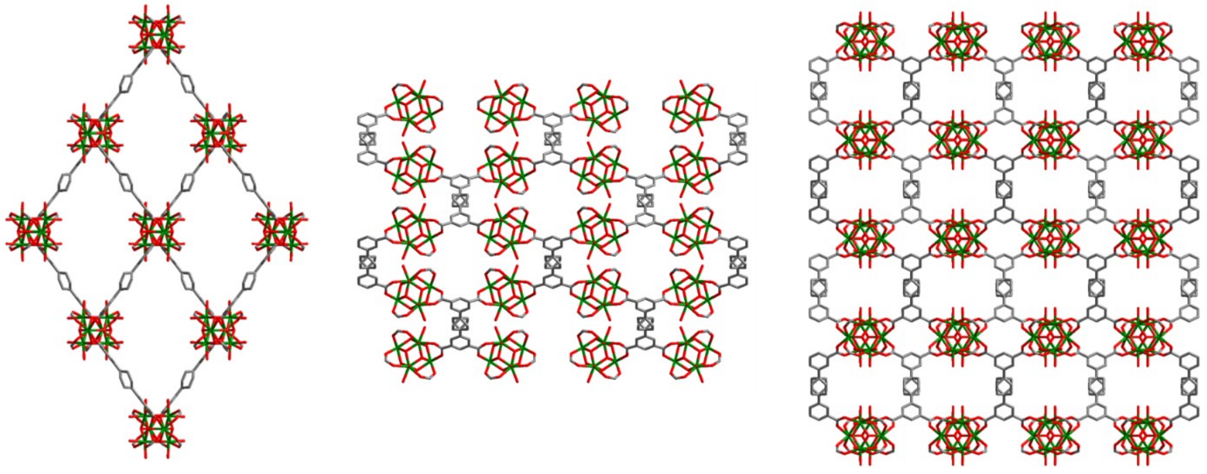

Figure S1. The single crystal structure of NU-1400 viewed along *a* (left), *b* (middle) and *c* (right) axis.[5]

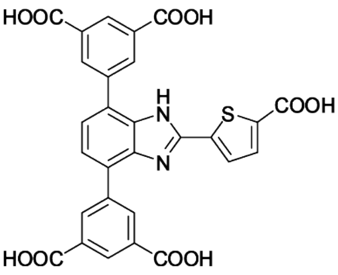

H_5CTBII

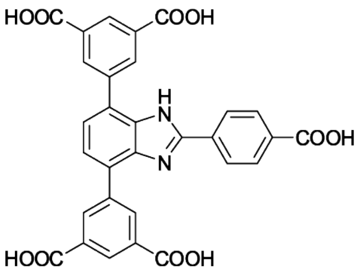

H_5CBII

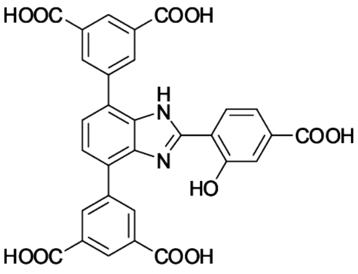

H_5CHBII

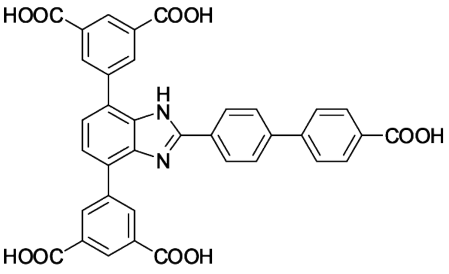

H_5CYBII

Figure S2. The pentacarboxylic acids reported in this work.

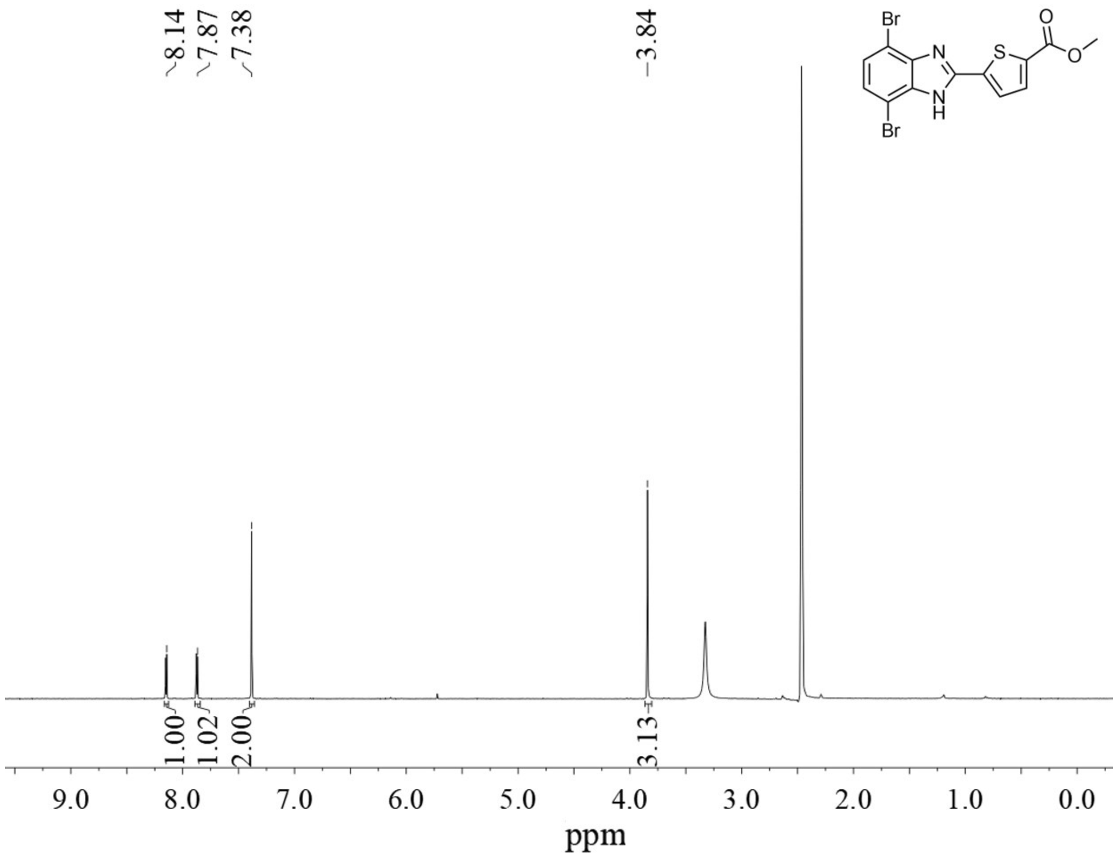

Figure S3. ^{1}H NMR spectrum of methyl 5-(4,7-dibromo-1H-benzo[d]imidazol-2-yl) thiophene-2-carboxylate in DMSO-d_6.

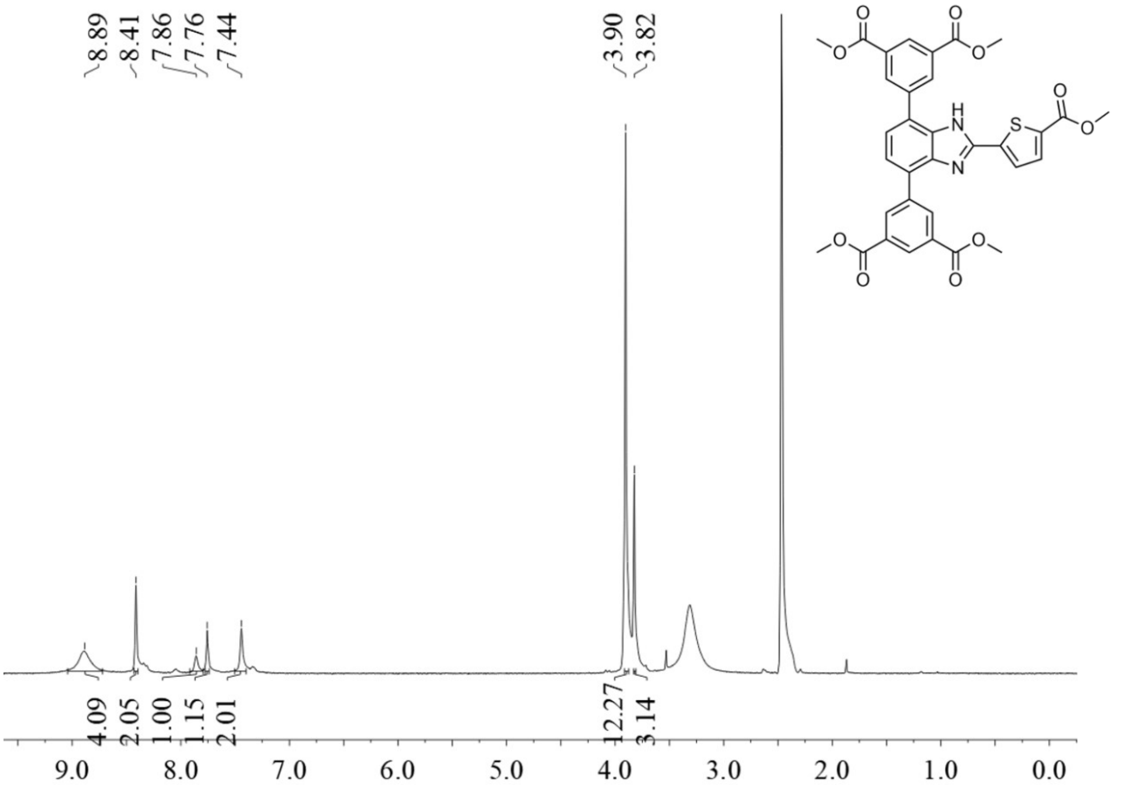

Figure S4. ^{1}H NMR spectrum of tetramethyl 5,5'-(2-(5-(methoxycarbonyl)thiophen-2-yl)-1H-benzo[d]imidazole-4,7-diyl)diisophthalate in DMSO-d_6.

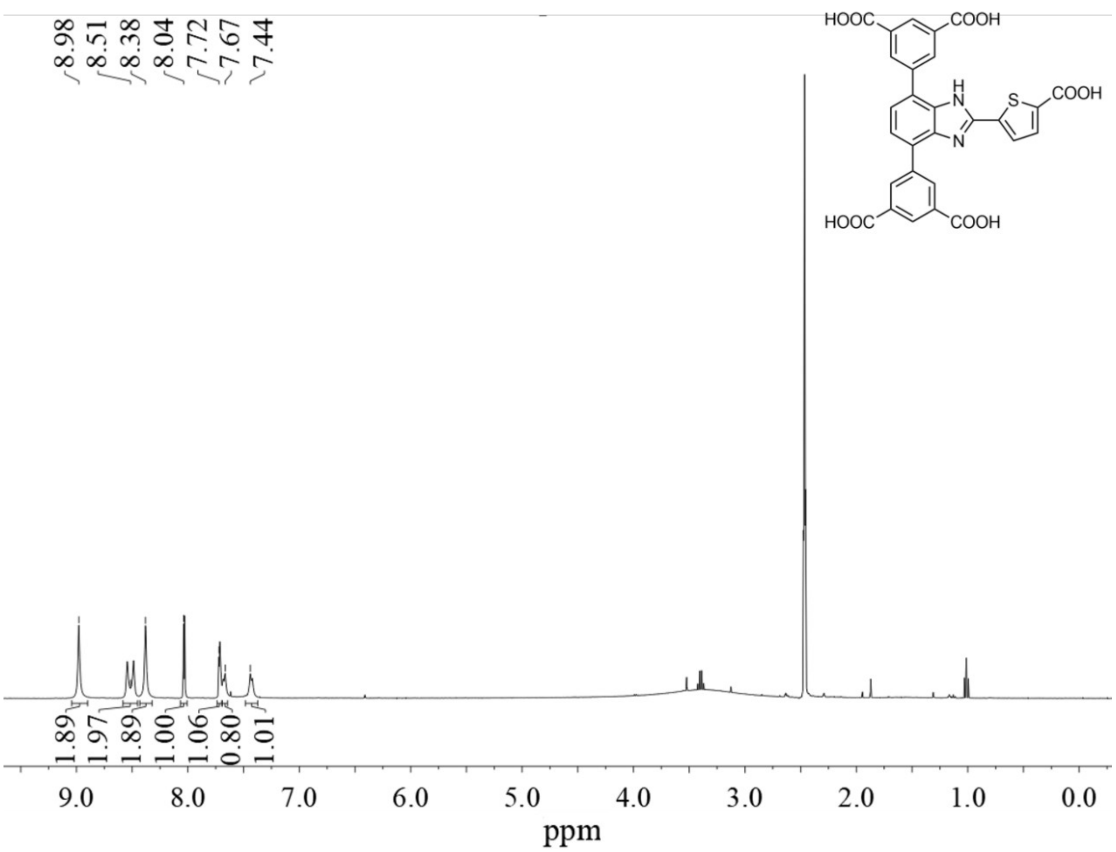

Figure S5. ^{1}H NMR spectrum of 5,5'-(2-(5-carboxythiophen-2-yl)-1H-benzo[d] imidazole-4,7-diyl)diisophthalic acid in DMSO-d_6.

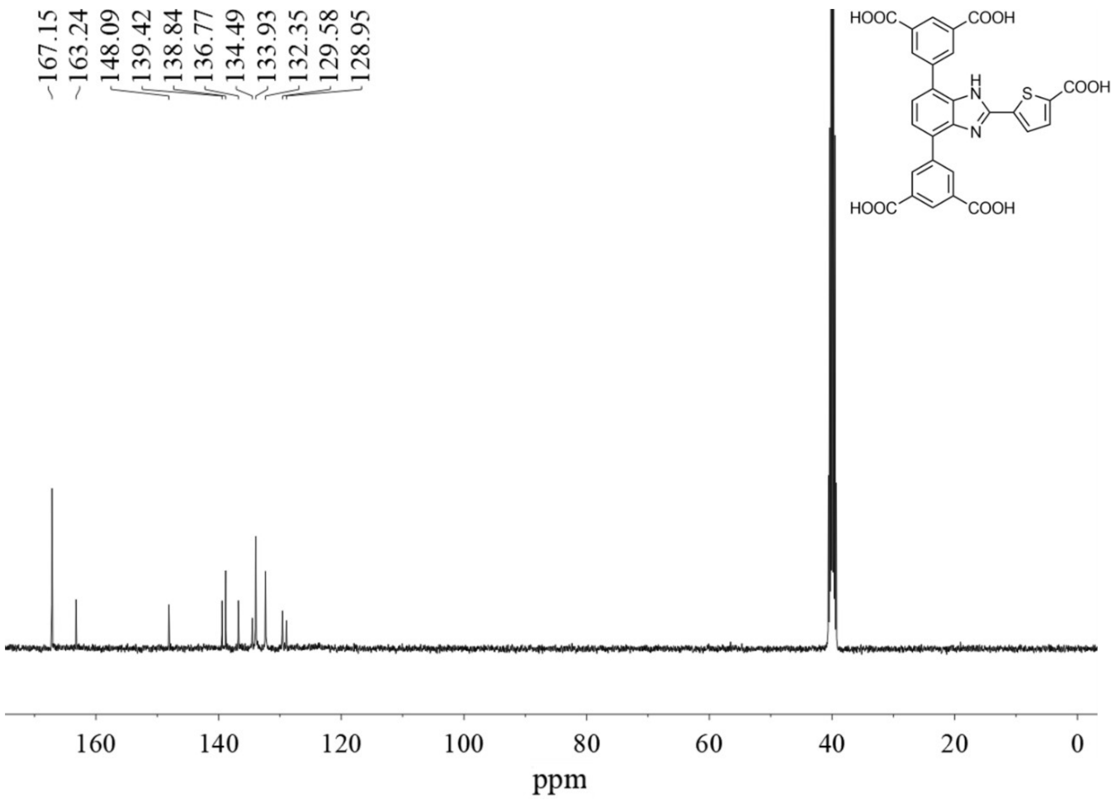

Figure S6. ^{13}C NMR spectrum of 5,5'-(2-(5-carboxythiophen-2-yl)-1H-benzo[d] imidazole-4,7-diyl)diisophthalic acid in DMSO-d_6.

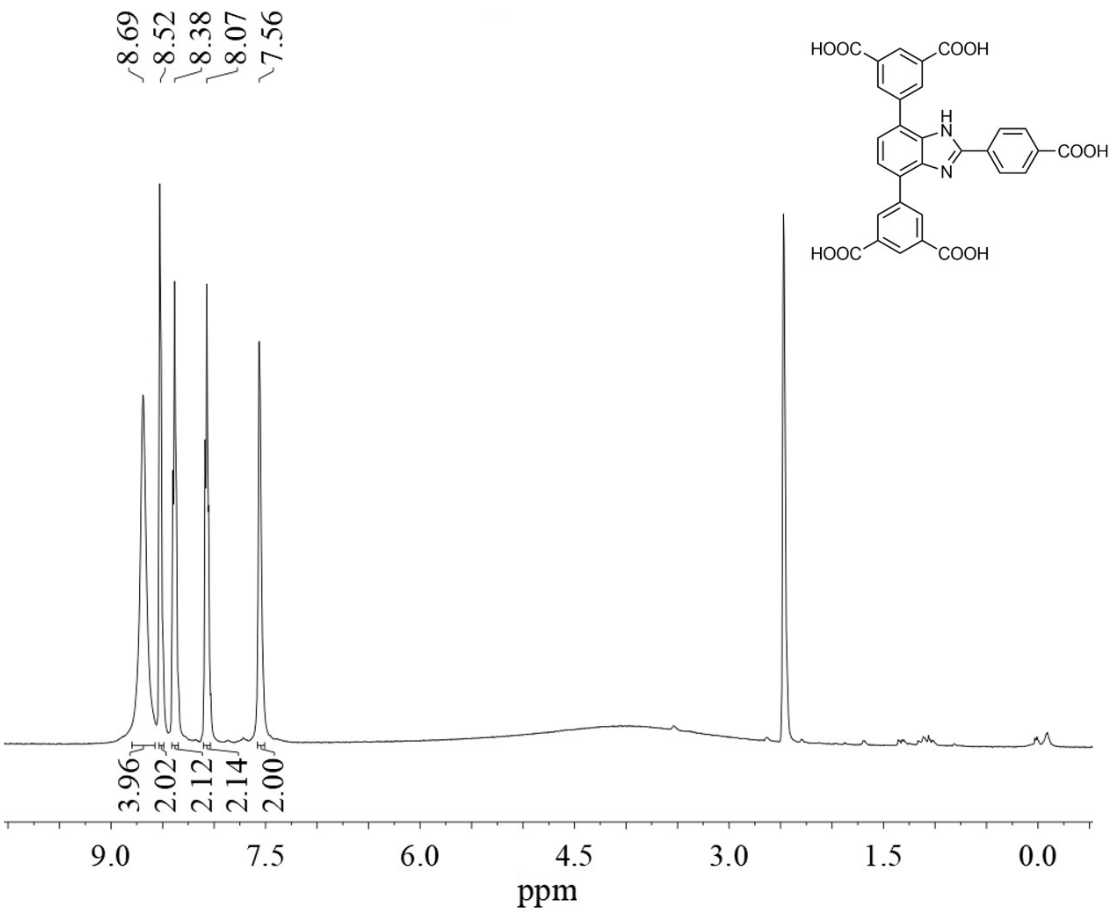

Figure S7. ^{1}H NMR spectrum of 5,5'-(2-(4-carboxyphenyl)-1H-benzo[d]imidazole-4,7-diyl) diisophthalic acid in DMSO-d_6.

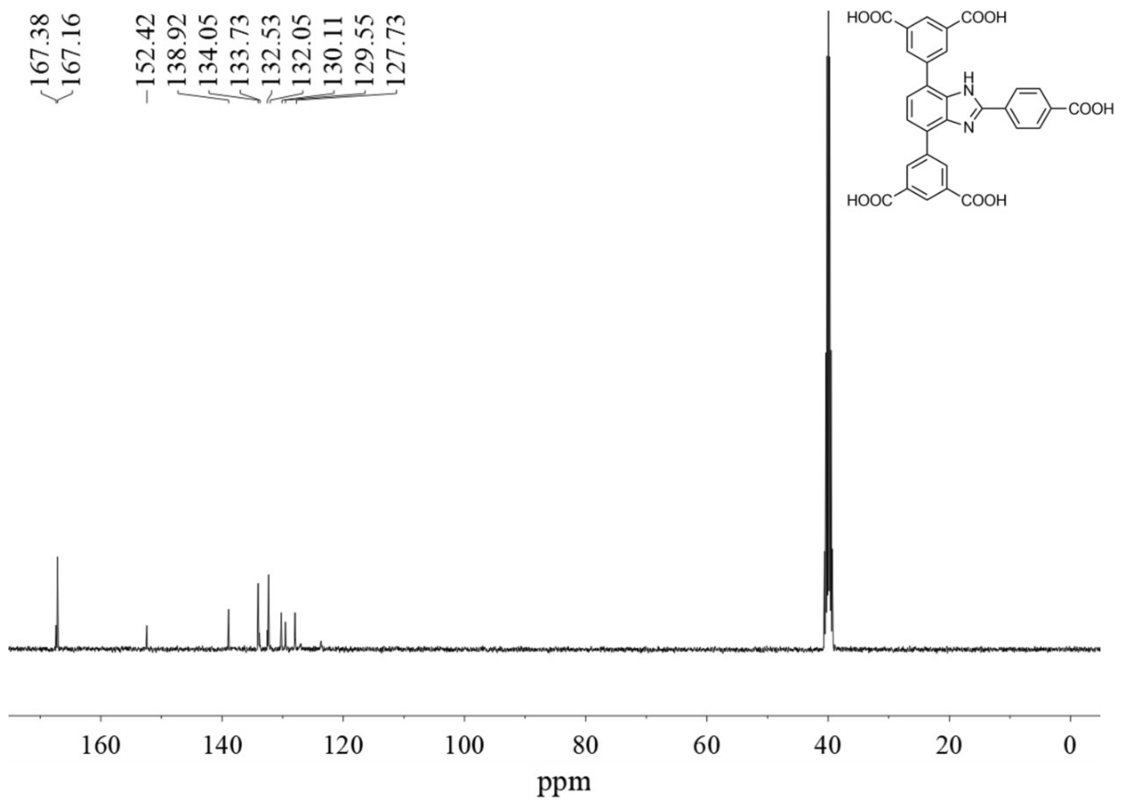

Figure S8. ^{13}C NMR spectrum of 5,5'-(2-(4-carboxyphenyl)-1H-benzo[d]imidazole-4,7-diyl) diisophthalic acid in DMSO-d_6.

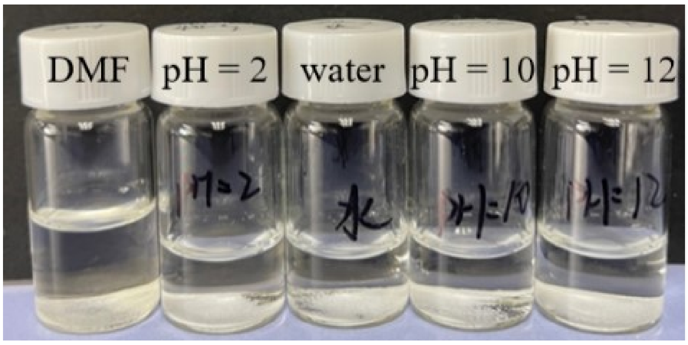

Figure S9. The images of HIAM-4040 samples in different solutions.

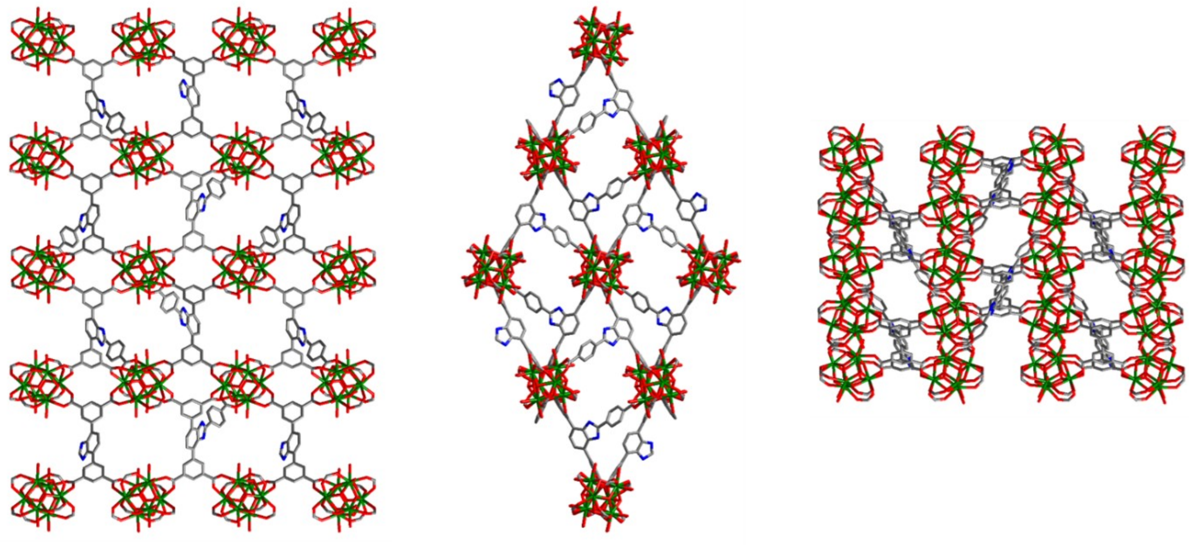

Figure S10. The single crystal structure of HIAM-4040 viewed along *a* (left), *b* (middle) and *c* (right) axis.

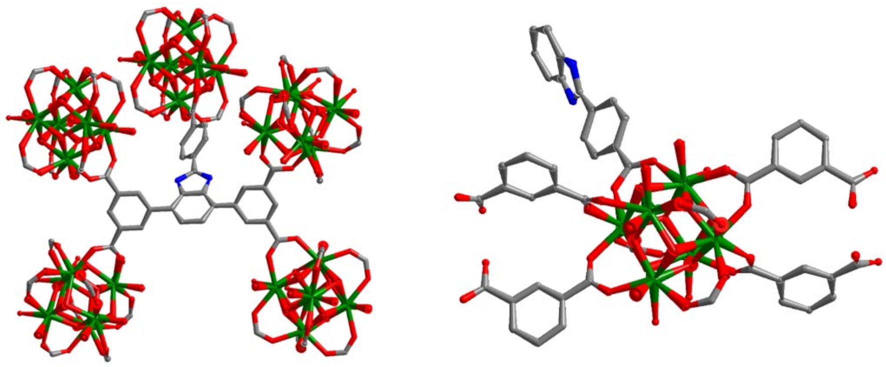

Figure S11. The connection model of H_5CBII (left) and the 5-connected Zr_6 cluster (right) in HIAM-4040.

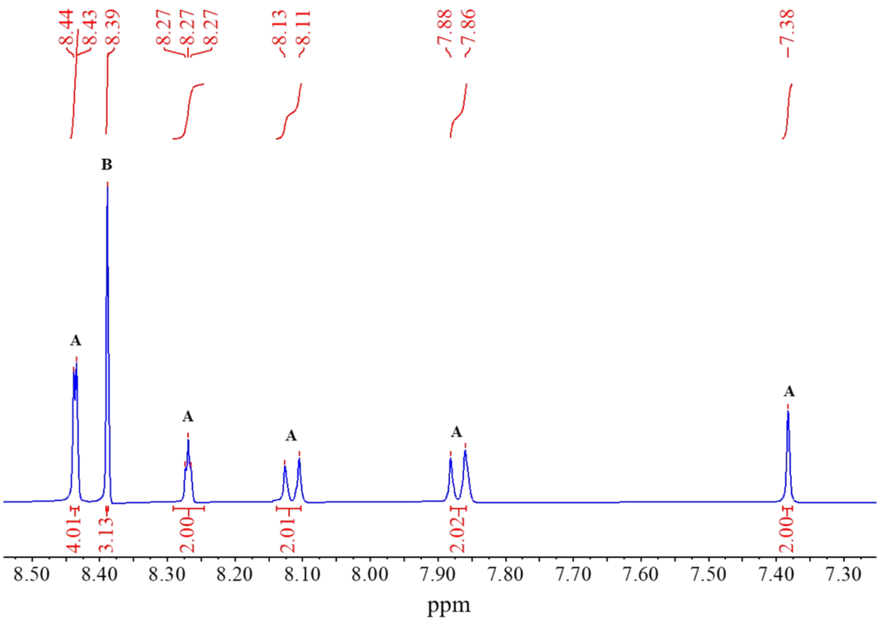

Figure S12. ^{1}H NMR spectrum of digested HIAM-4040 in D_2O. Peaks labeled with "A" and "B" are from CBII and formate, respectively. The ratio of CBII and formate is 1:3, which matches well with data from the single-crystal structure.

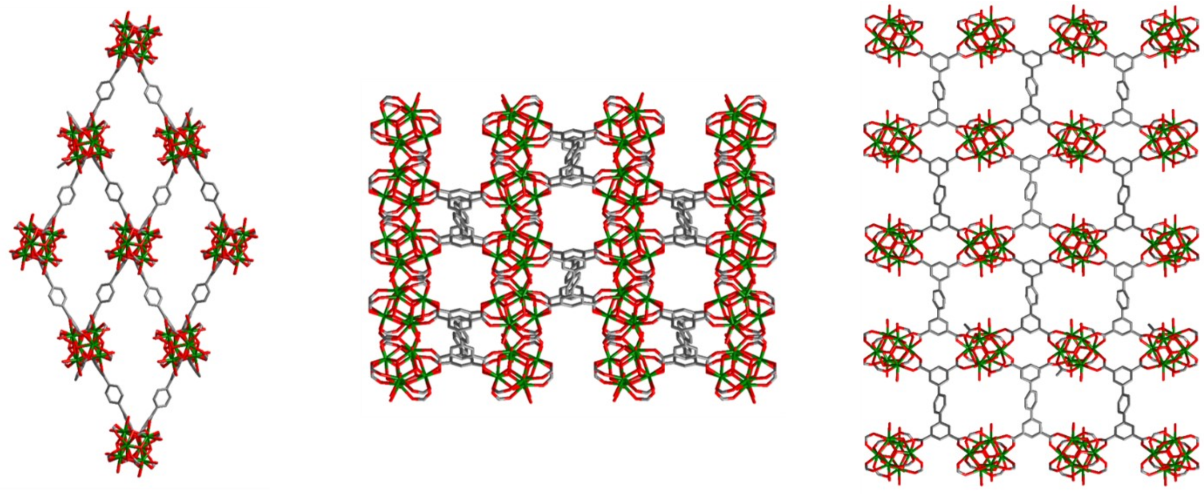

Figure S13. The structure adopted from HIAM-4040 after deleting 4-(1H-imidazol-2-yl)benzoic acid group viewed along *b* (left), *c* (middle) and *a* (right) axis.

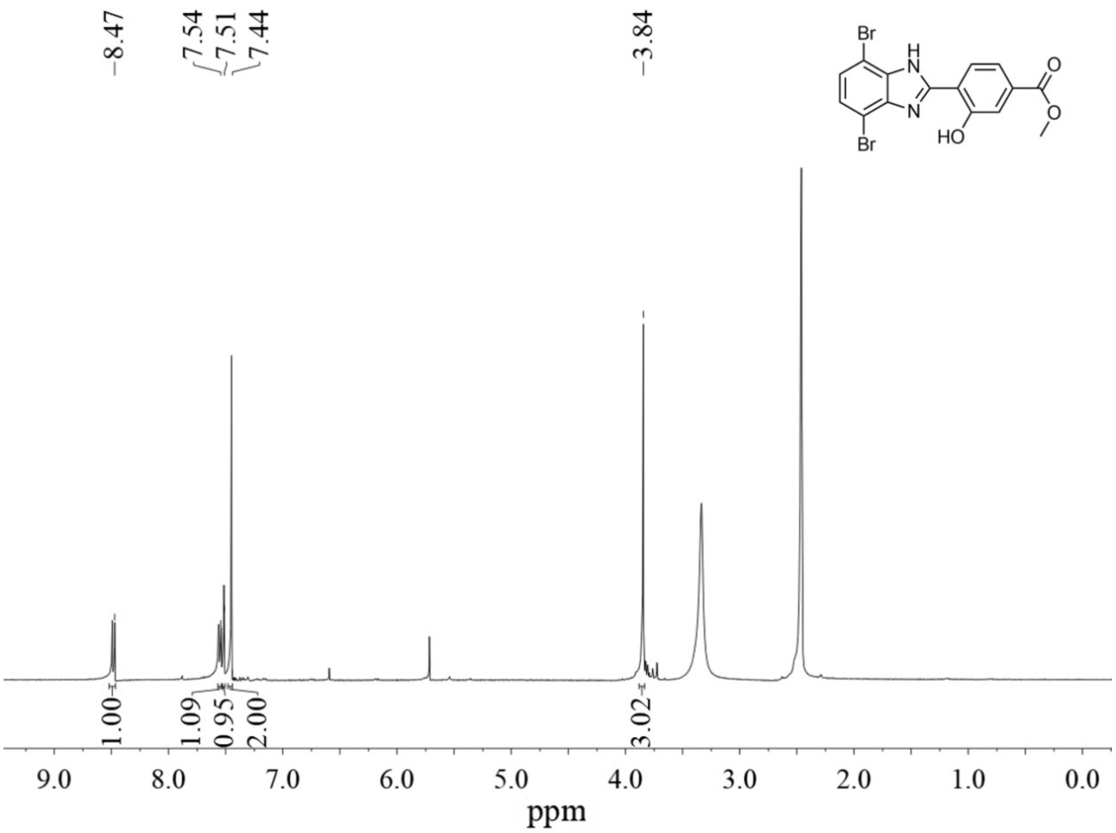

Figure S14. ^{1}H NMR spectrum of methyl 4-(4,7-dibromo-1H-benzo[d]imidazol-2-yl) -3-hydroxybenzoate in DMSO-d_6.

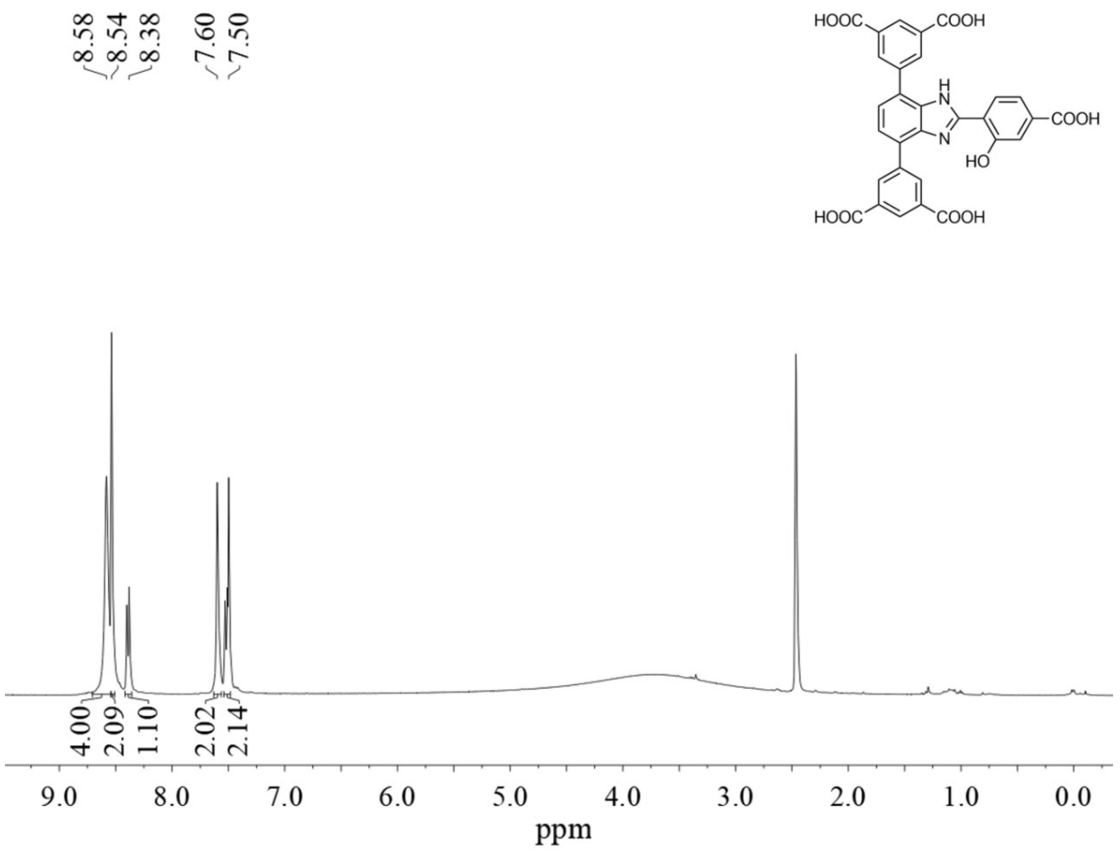

Figure S15. ^{1}H NMR spectrum of 5,5'-(2-(4-carboxy-2-hydroxyphenyl)-1H-benzo [d]imidazole-4,7-diyl) diisophthalic acid in DMSO-d_6.

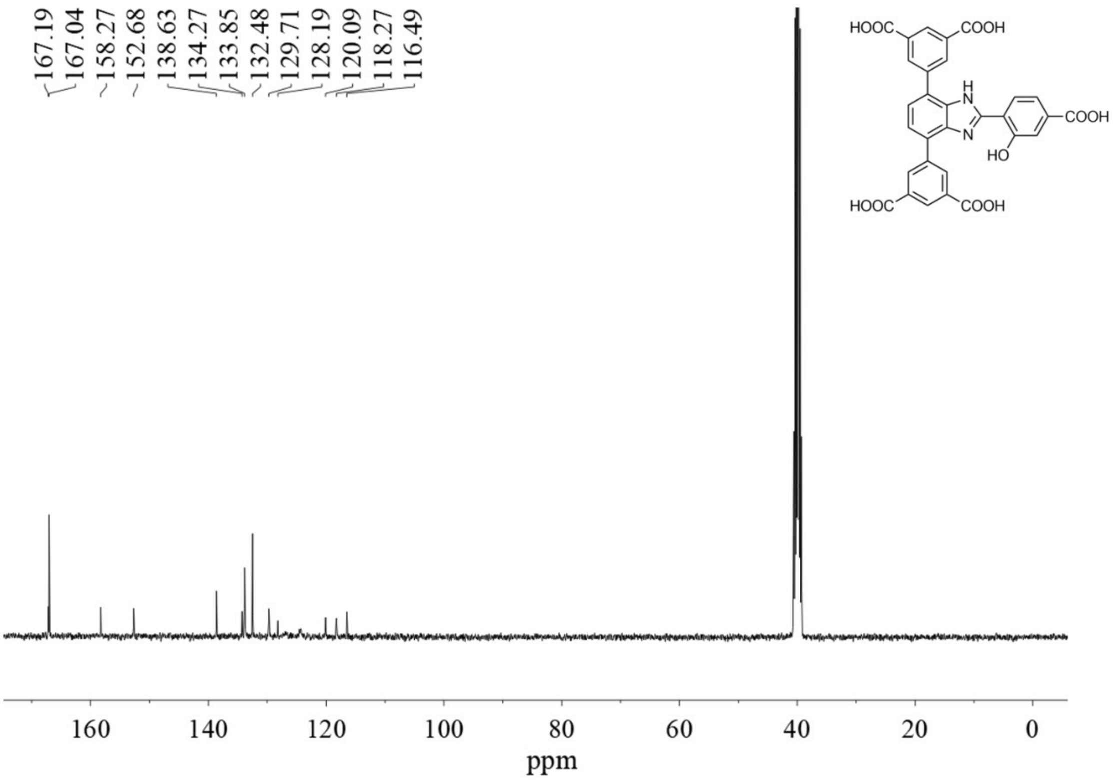

Figure S16. ^{13}C NMR spectrum of 5,5'-(2-(4-carboxy-2-hydroxyphenyl)-1H-benzo [d]imidazole-4,7-diyl) diisophthalic acid in DMSO-d_6.

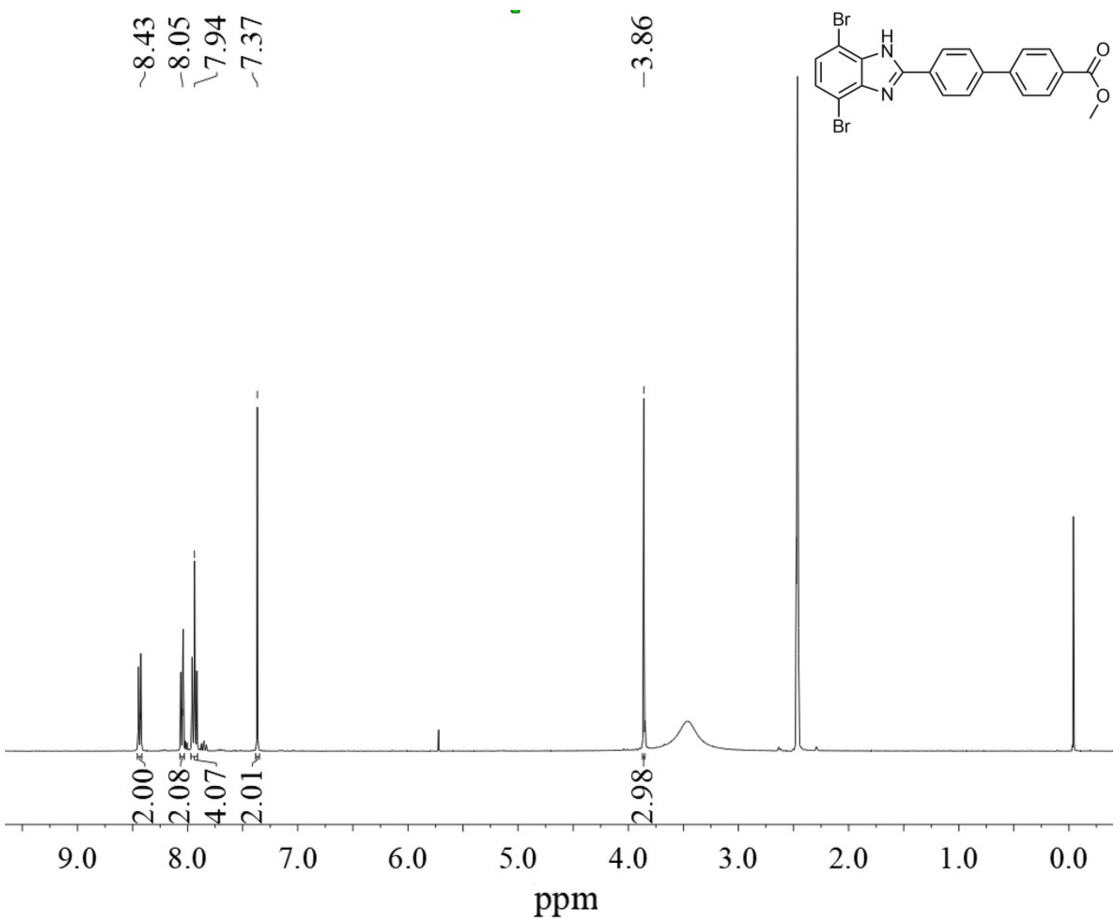

Figure S17. ¹H NMR spectrum of methyl 4'-(4,7-dibromo-1H-benzo[d]imidazol-2-yl) biphenyl-4-carboxylate in DMSO-d_6.

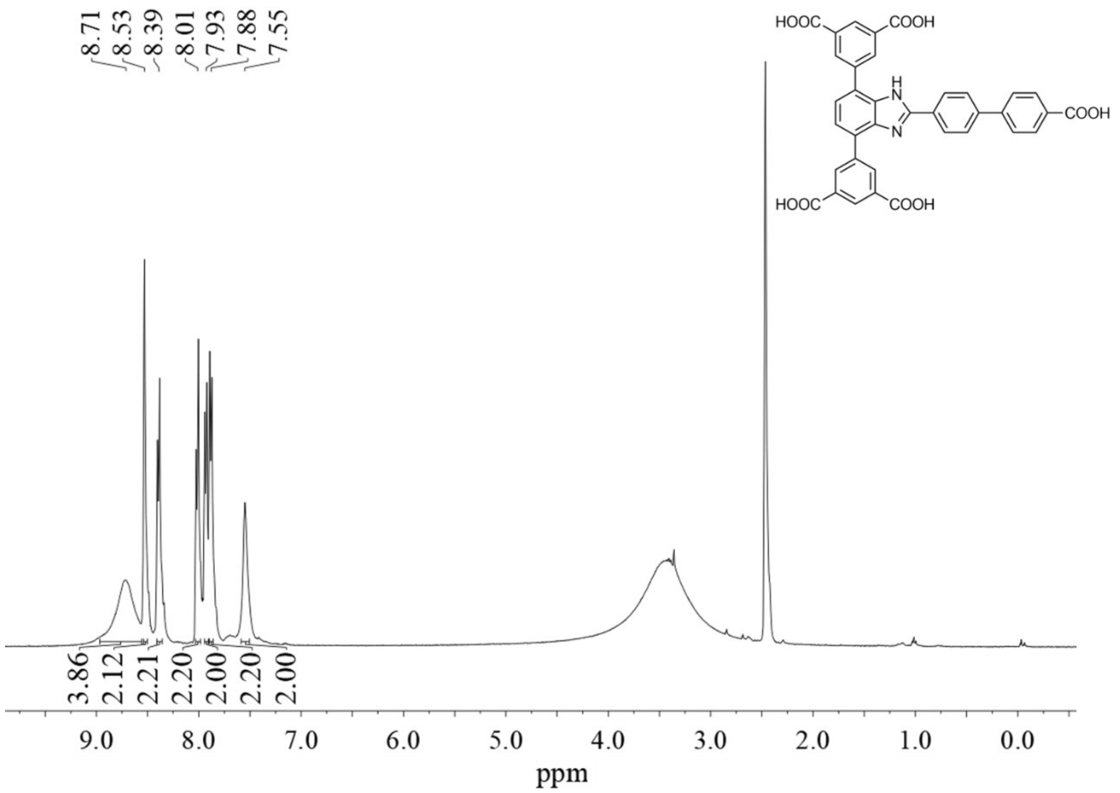

Figure S18. ^{1}H NMR spectrum of 5,5'-(2-(4'-carboxybiphenyl-4-yl)-1H-benzo[d] imidazole-4,7-diyl) diisophthalic acid in DMSO-d_6.

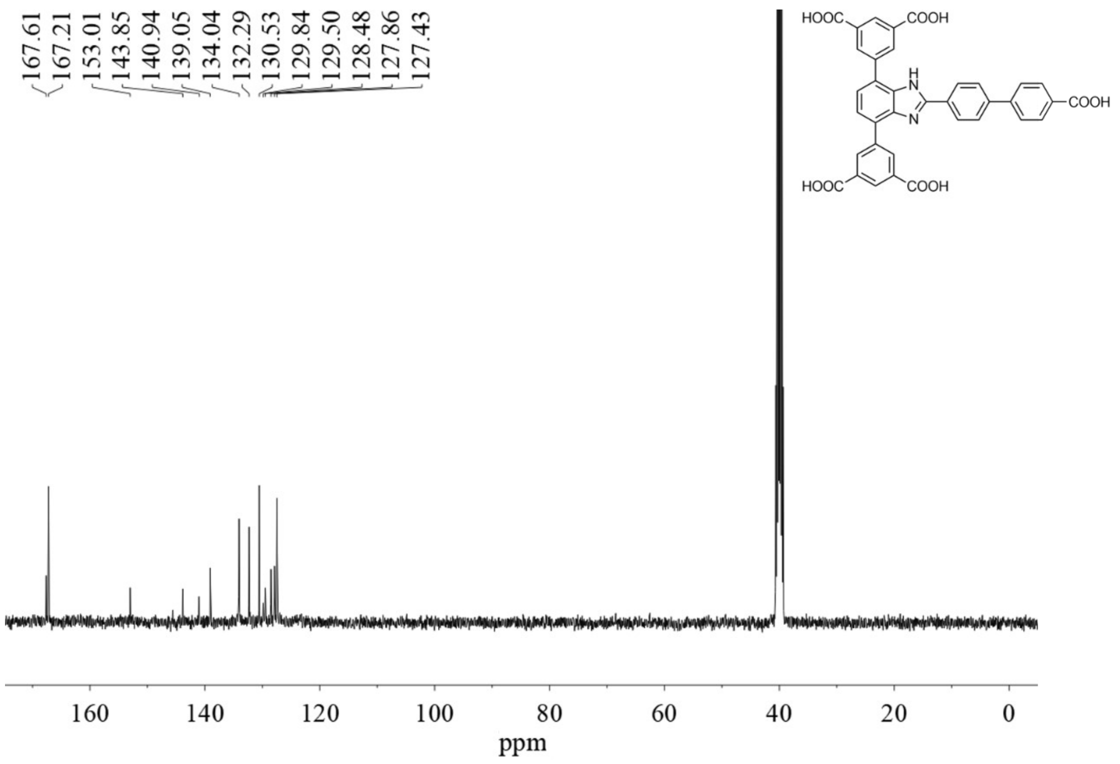

Figure S19. ^{13}C NMR spectrum of 5,5'-(2-(4'-carboxybiphenyl-4-yl)-1H-benzo[d] imidazole-4,7-diyl) diisophthalic acid in DMSO-d_6.

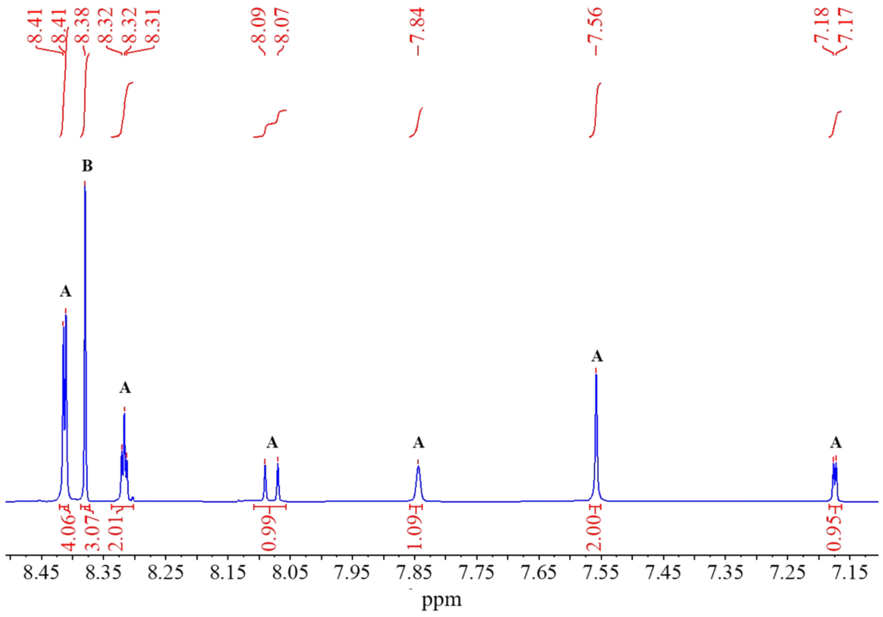

Figure S20. ^{1}H NMR spectrum of digested HIAM-4040-OH in D_2O. Peaks labeled with "A" and "B" are from CHBII and formate, respectively. The ratio of CHBII and formate is 1:3, which matches well with data from the single-crystal structure.

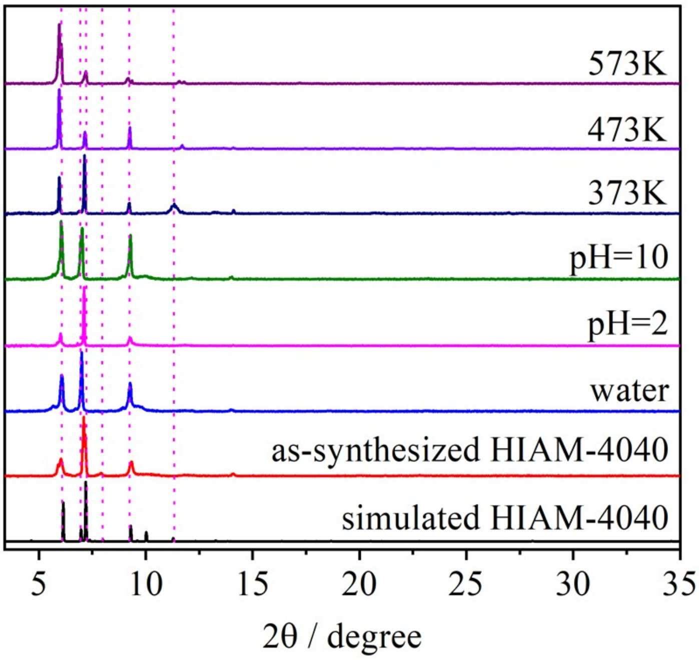

Figure S21. The PXRD patterns of HIAM-4040 after treatment under various conditions for 24 hours.

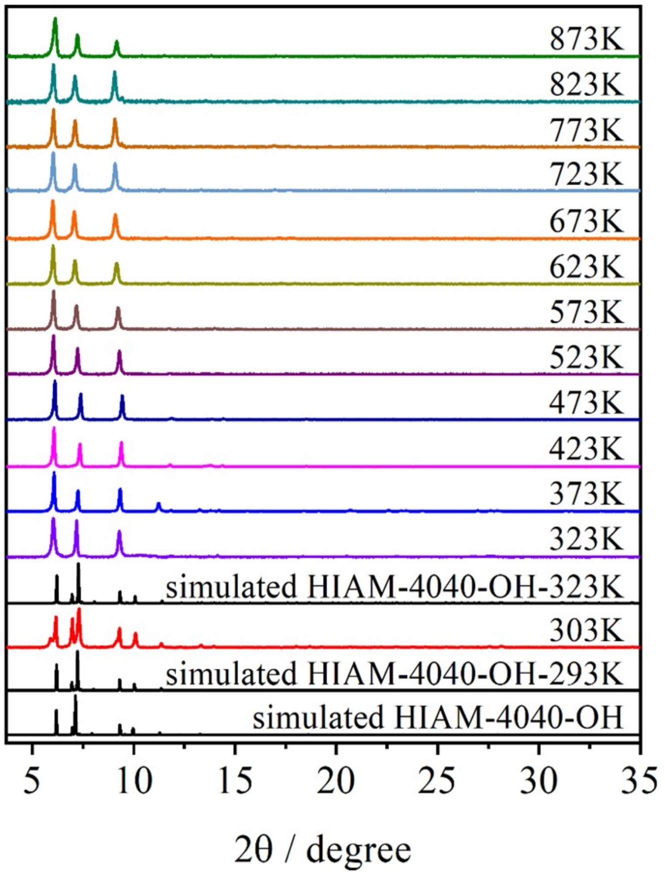

Figure S22. The temperature-dependent PXRD patterns of HIMA-4040-OH.

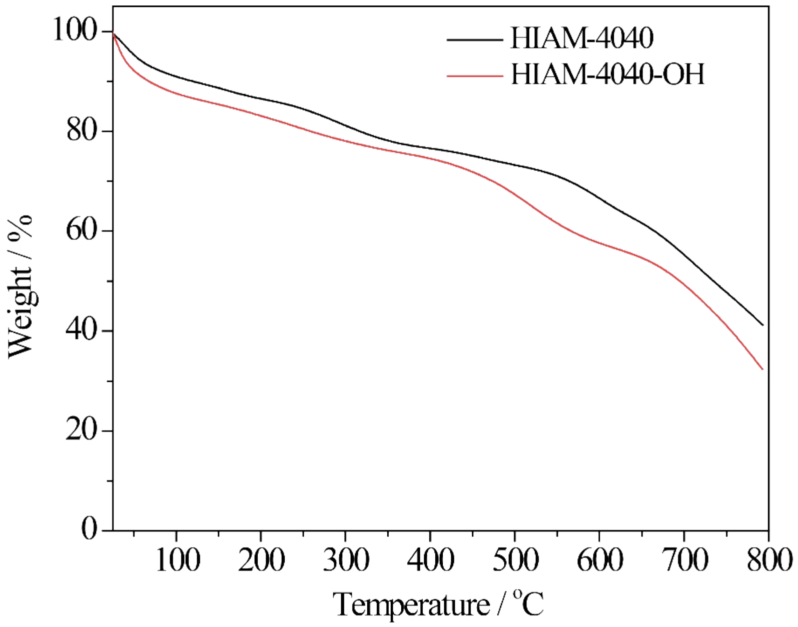

Figure S23. The TG curves of HIAM-4040 and HIAM-4040-OH.

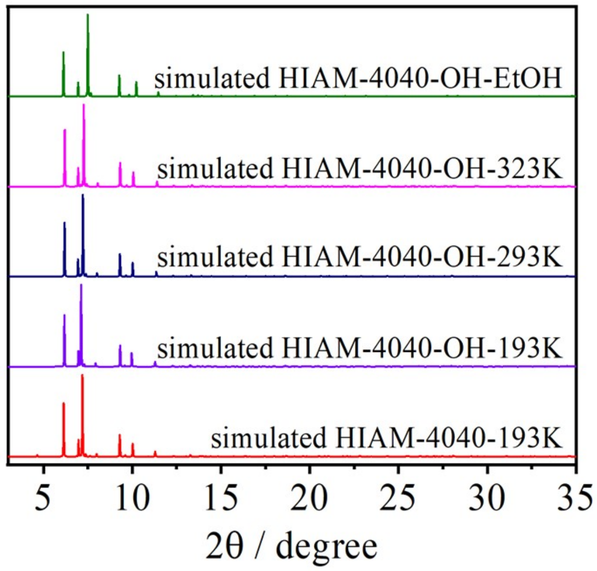

Figure S24. The PXRD patterns of simulated HIAM-4040-193K, HIAM-4040-OH-193K, HIAM-4040-OH-293K, HIAM-4040-OH-323K and HIAM-4040-OH-EtOH.

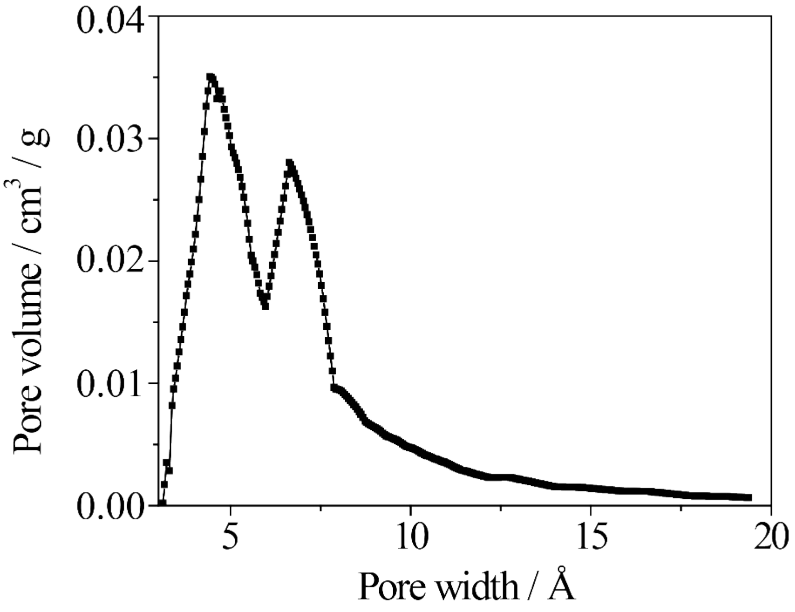

Figure S25. The pore size distribution profile of HIAM-4040-OH.

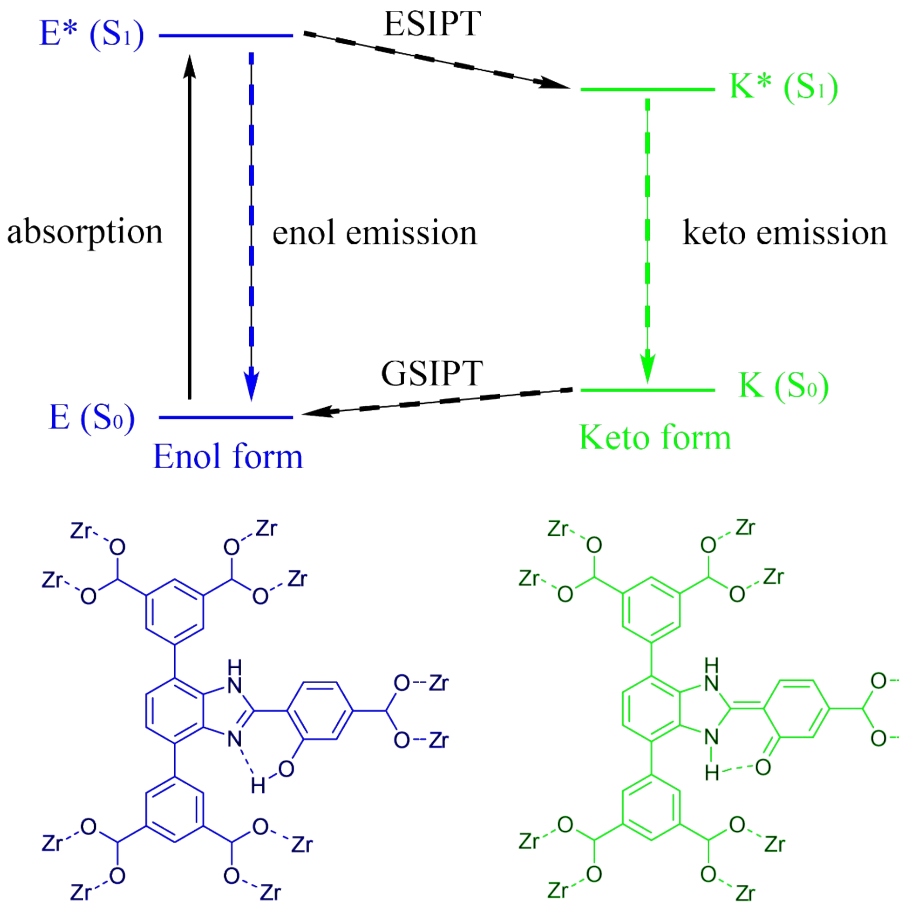

Figure S26. The diagram of ESIPT process in HIAM-4040-OH. The enol form (left) and the keto form (right) of CHBII in HIAM-4040-OH and the corresponding energy levels.[6-7]

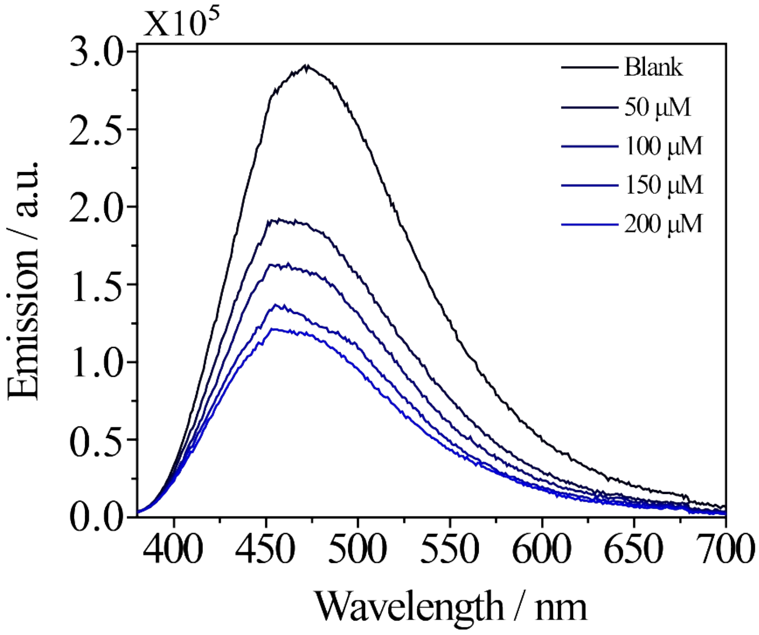

Figure S27. The emission spectra of HIAM-4040 with increased concentration of HClO.

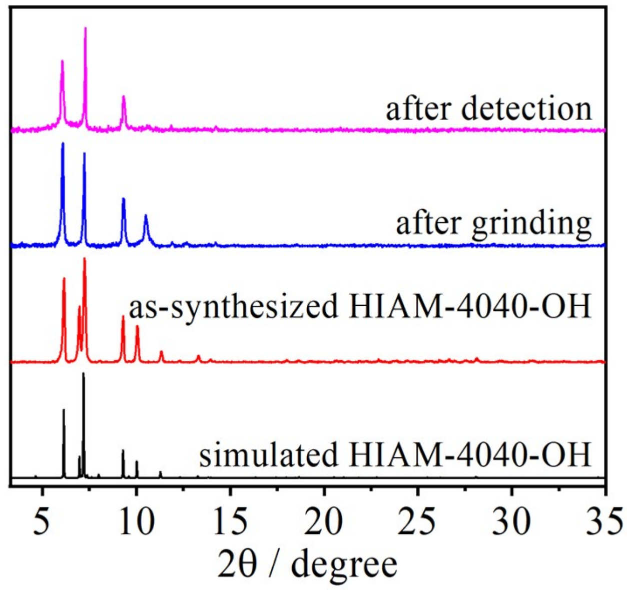

Figure S28. The PXRD patterns of simulated HIAM-4040-OH, as-synthesized HIAM-4040-OH, HIAM-4040-OH after grinding and detection of HClO.

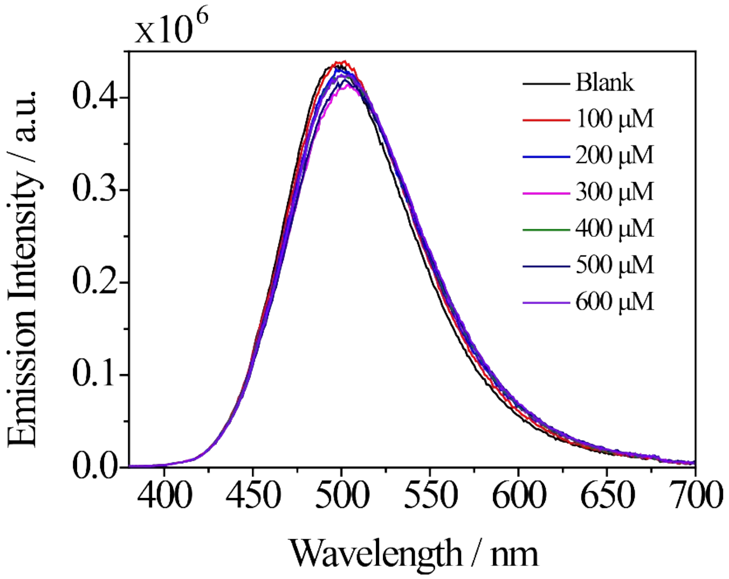

Figure S29. The emission spectra of HIAM-4040-OH with gradually increased concentration of HCl.

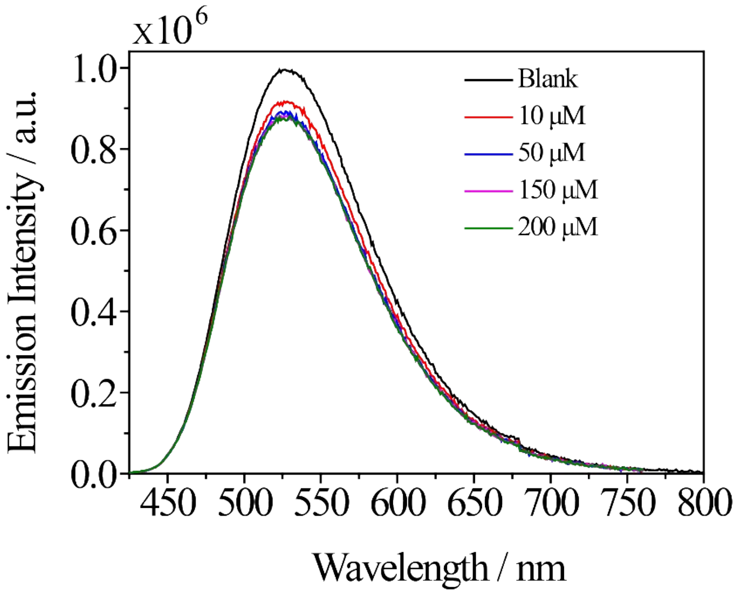

Figure S30. The emission spectra of HIAM-4011 with gradually increased concentration of HClO.

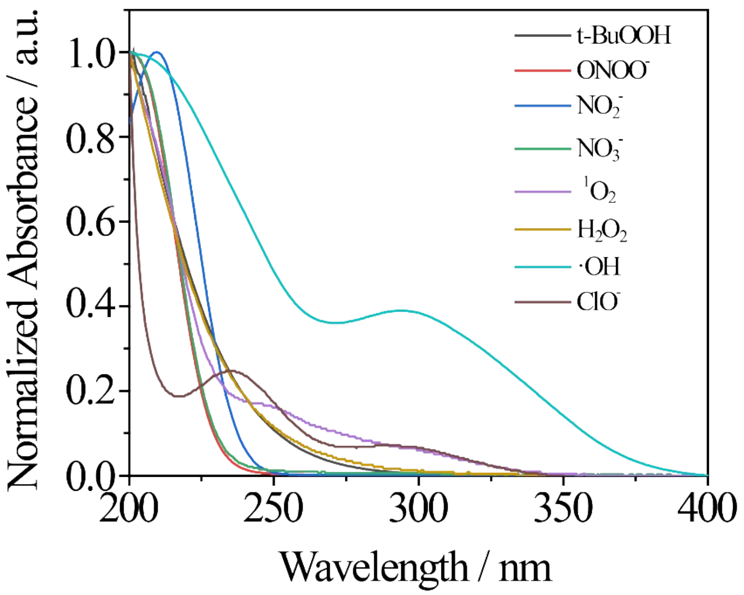

Figure S31. The UV-vis absorption spectra of different kinds of ROS and RNS.

Table S1. Crystal data and structure refinement for HIAM-4040.

CCDC No.	2274419	
Empirical formula	$C_{33}H_{15}N_2O_{32}Zr_6$	
Formula weight	1498.79	
Temperature	193.0	
Wavelength	1.34139 Å	
Crystal system	orthorhombic	
Space group	$P2_12_12_1$	
Unit cell dimensions	a = 13.5866(8) Å	α = 90
	b = 25.3514(14) Å	β = 90
	c = 28.7868(15) Å	γ = 90
Volume	9915.3(10) $Å^3$	
Z	4	
Density (calculated)	1.004 g/cm^3	
Absorption coefficient	3.645 mm^{-1}	
F(000)	2892.0	
Crystal size	0.13 x 0.12 x 0.1 mm^3	
Theta range for data collection	4.04 to 120.638°	
Index ranges	-15<=h<=16, -30<=k<=32, -31<=l<=36	
Reflections collected	76214	
Independent reflections	20916 [R(int) = 0.0912, R(sigma) = 0.0690]	
Data / restraints / parameters	20916 / 0 / 659	
Goodness-of-fit on F^2	0.913	
Final R indices [I>2sigma(I)]	R1 = 0.0383, wR2 = 0.0878	
R indices (all data)	R1 = 0.0512, wR2 = 0.0920	
Largest diff. peak and hole	0.44 and -0.65 e.$Å^{-3}$	

Table S2. Crystal data and structure refinement for HIAM-4040-OH-193K.

CCDC No.	2290437	
Empirical formula	$C_{33}H_{16}N_2O_{33}Zr_6$	
Formula weight	1515.80	
Temperature	193.0	
Wavelength	0.71073 Å	
Crystal system	orthorhombic	
Space group	Pnma	
Unit cell dimensions	a = 28.6081(13) Å	α = 90
	b = 25.3758(13) Å	β = 90
	c = 13.7747(7) Å	γ = 90
Volume	9999.8(9) $Å^3$	
Z	4	
Density (calculated)	1.007 g/cm^3	
Absorption coefficient	0.653 mm^{-1}	
F(000)	2928.0	
Crystal size	0.13 x 0.12 x 0.1 mm^3	
Theta range for data collection	3.364 to 55.028°	
Index ranges	-37<=h<=23, -32<=k<=20, -17<=l<=17	
Reflections collected	43414	
Independent reflections	11462[R(int) = 0.1139, R(sigma)=0.1083]	
Data / restraints / parameters	11462 / 130 / 440	
Goodness-of-fit on F2	1.059	
Final R indices [I>2sigma(I)]	R1 = 0.0734, wR2 = 0.1763	
R indices (all data)	R1 = 0.1123, wR2 = 0.1972	
Largest diff. peak and hole	0.89 and -0.59 e.$Å^{-3}$	

Table S3. Crystal data and structure refinement for HIAM-4040-OH-293K.

CCDC No.	2290440	
Empirical formula	$C_{33}H_{15}N_2O_{33}Zr_6$	
Formula weight	1514.79	
Temperature	293.0	
Wavelength	1.54178 Å	
Crystal system	orthorhombic	
Space group	Pnma	
Unit cell dimensions	a = 28.5667(11) Å	α = 90
	b = 25.4597(8) Å	β = 90
	c = 13.5472(7) Å	γ = 90
Volume	9852.9(7) $Å^3$	
Z	4	
Density (calculated)	1.021 g/cm^3	
Absorption coefficient	5.487 mm^{-1}	
F(000)	2924.0	
Crystal size	0.13 x 0.12 x 0.1 mm^3	
Theta range for data collection	7.392 to 159.992°	
Index ranges	$-35 \le h \le 34$, $-31 \le k \le 30$, $-16 \le l \le 16$	
Reflections collected	85833	
Independent reflections	10689[R(int) = 0.0821, R(sigma)=0.0472]	
Data / restraints / parameters	10689 / 94 / 451	
Goodness-of-fit on F2	1.078	
Final R indices [I>2sigma(I)]	R1 = 0.0431, wR2 = 0.1264	
R indices (all data)	R1 = 0.0563, wR2 = 0.1342	
Largest diff. peak and hole	1.06 and -0.86 $e.Å^{-3}$	

Table S4. Crystal data and structure refinement for HIAM-4040-OH-323K.

CCDC No.	2290438	
Empirical formula	$C_{33}H_{16}N_2O_{33}Zr_6$	
Formula weight	1515.80	
Temperature	323.0	
Wavelength	1.54178 Å	
Crystal system	orthorhombic	
Space group	Pnma	
Unit cell dimensions	a = 28.527(6) Å	α = 90
	b = 25.427(4) Å	β = 90
	c = 13.449(3) Å	γ = 90
Volume	9756(3) $Å^3$	
Z	4	
Density (calculated)	1.032 g/cm^3	
Absorption coefficient	5.542 mm^{-1}	
F(000)	2928.0	
Crystal size	0.13 x 0.12 x 0.1 mm^3	
Theta range for data collection	7.106 to 137.334°	
Index ranges	$-34 \leq h \leq 33$, $-30 \leq k \leq 30$, $-16 \leq l \leq 13$	
Reflections collected	52670	
Independent reflections	9188 [R(int) = 0.1082, R(sigma) =0.0765]	
Data / restraints / parameters	9188 / 173 / 445	
Goodness-of-fit on F2	0.930	
Final R indices [I>2sigma(I)]	R1 = 0.0694, wR2 = 0.1860	
R indices (all data)	R1 = 0.0928, wR2 = 0.2078	
Largest diff. peak and hole	1.24 and -0.99 e.$Å^{-3}$	

Table S5. Crystal data and structure refinement for HIAM-4040-OH-EtOH.

CCDC No.	2290436	
Empirical formula	$C_{33}H_{20}N_2O_{27}Zr_6$	
Formula weight	1423.83	
Temperature	193.0	
Wavelength	0.71073 Å	
Crystal system	orthorhombic	
Space group	Imma	
Unit cell dimensions	a = 25.437(2) Å	α = 90
	b = 28.850(2) Å	β = 90
	c = 12.9217(11) Å	γ = 90
Volume	9482.6(13) $Å^3$	
Z	4	
Density (calculated)	0.997 g/cm^3	
Absorption coefficient	0.680 mm^{-1}	
F(000)	2752.0	
Crystal size	0.13 x 0.12 x 0.1 mm^3	
Theta range for data collection	3.536 to 52.786°	
Index ranges	$-31 \le h \le 31$, $-36 \le k \le 34$, $-16 \le l \le 15$	
Reflections collected	37167	
Independent reflections	5072 [R(int) = 0.1322, R(sigma) =0.0831]	
Data / restraints / parameters	5072 / 196 / 293	
Goodness-of-fit on F2	1.040	
Final R indices [I>2sigma(I)]	R1 = 0.1030, wR2 = 0.2936	
R indices (all data)	R1 = 0.1433, wR2 = 0.3269	
Largest diff. peak and hole	1.24 and -0.69 e.$Å^{-3}$	

Table S6. Crystal data and structure refinement for HIAM-4040-OH-pH2.

CCDC No.	2290439	
Empirical formula	$C_{33}H_{16}N_2O_{33}Zr_6$	
Formula weight	1515.80	
Temperature	193.0	
Wavelength	0.71073 Å	
Crystal system	orthorhombic	
Space group	Pnma	
Unit cell dimensions	a = 28.822(3) Å	α = 90
	b = 25.209(2) Å	β = 90
	c = 13.8069(13) Å	γ = 90
Volume	10031.9(15) Å^3	
Z	4	
Density (calculated)	1.004 g/cm^3	
Absorption coefficient	0.651 mm^{-1}	
F(000)	2928.0	
Crystal size	0.13 x 0.12 x 0.1 mm^3	
Theta range for data collection	3.648 to 52.748°	
Index ranges	-35<=h<=36, -31<=k<=31, -17<=l<=14	
Reflections collected	82826	
Independent reflections	10477[R(int) = 0.1338, R(sigma)=0.0767]	
Data / restraints / parameters	10477 / 215 / 454	
Goodness-of-fit on F2	1.038	
Final R indices [I>2sigma(I)]	R1 = 0.0524, wR2 = 0.1387	
R indices (all data)	R1 = 0.0816, wR2 = 0.1522	
Largest diff. peak and hole	0.60 and -0.64 e.Å^{-3}	

Table S7. Crystal data and structure refinement for HIAM-4040-OH-pH12.

CCDC No.	2290441	
Empirical formula	$C_{33}H_{32}N_2O_{33}Zr_6$	
Formula weight	1531.92	
Temperature	193.0	
Wavelength	0.71073 Å	
Crystal system	orthorhombic	
Space group	Pnma	
Unit cell dimensions	a = 28.8021(13) Å	α = 90
	b = 25.3660(12) Å	β = 90
	c = 13.6242(7) Å	γ = 90
Volume	9953.8(8) $Å^3$	
Z	4	
Density (calculated)	1.022 g/cm^3	
Absorption coefficient	0.656 mm^{-1}	
F(000)	2992.0	
Crystal size	0.13 x 0.12 x 0.1 mm^3	
Theta range for data collection	3.394 to 54.964°	
Index ranges	-34<=h<=37, -32<=k<=32, -17<=l<=16	
Reflections collected	89777	
Independent reflections	11639[R(int) = 0.1112, R(sigma)=0.0631]	
Data / restraints / parameters	11639 / 106 / 430	
Goodness-of-fit on F2	1.058	
Final R indices [I>2sigma(I)]	R1 = 0.0513, wR2 = 0.1377	
R indices (all data)	R1 = 0.0741, wR2 = 0.1485	
Largest diff. peak and hole	0.93 and -0.54 e.$Å^{-3}$	

Table S8. Summary of the cell parameters for HIAM-4040-193K, HIAM-4040-OH-193K, HIAM-4040-OH-293K, HIAM-4040-OH-323K, HIAM-4040-OH-EtOH, HIAM-4040-OH-pH2 and HIAM-4040-OH-pH12.

	a/Å	b/Å	c/Å
HIAM-4040-193K	13.5866(8)	25.3514(14)	28.7868(15)
HIAM-4040-OH-193K	28.6081(13)	25.3758(13)	13.7747(7)
HIAM-4040-OH-293K	28.5667(11)	25.4597(8)	13.5472(7)
HIAM-4040-OH-323K	28.527(6)	25.427(4)	13.449(3)
HIAM-4040-OH-EtOH	25.437(2)	28.850(2)	12.9217(11)
HIAM-4040-OH-pH2	28.822(3)	25.209(2)	13.8069(13)
HIAM-4040-OH-pH12	28.8021(13)	25.3660(12)	13.6242(7)

MOFs	LOD (μM)	Solution	References
CD/CCM@ZIF-8	0.067	ethanol	8
UiO-68-ol	0.10	PBS	9
PDA/Eu/PDA-UiO-66- NH_2	0.10	water	10
UiO-68-PT	0.28	water	11
ZIF-8 based composites	6.70	buffer	12
$\{[Eu_2Cu(IN)_5(CO_3)(H_2O)]\}$	10.00	DMF	13
HIAM-4040-OH	1.57	water	this work

Table S9. Summary of MOF-based chemical sensors for HClO detection.

Table S10. Calculated and measured elemental analysis data for HIAM-4040.

MOF	C (%)	H (%)	N (%)	O (%)	Zr (%)

Calculated	HIAM-4040	25.35	2.05	1.85	33.80	36.95
Measured	HIAM-4040	27.86	7.90	3.00	26.40	34.84

Table S11. Calculated and measured elemental analysis data for HIAM-4040-OH.

	MOF	C (%)	H (%)	N (%)	O (%)	Zr (%)
Calculated	HIAM-4040-OH	25.07	2.09	1.83	34.47	36.54
Measured	HIAM-4040-OH	27.97	5.98	3.55	25.72	36.78

References

1. He, T.; Zhang, Y.-Z.; Kong, X.-J.; Yu, J.; Lv, X.-L.; Wu, Y.; Guo, Z.-J.; Li, J.-R., Zr(IV)-Based Metal-Organic Framework with T-Shaped Ligand: Unique Structure, High Stability, Selective Detection, and Rapid Adsorption of $Cr_2O_7^{2-}$ in Water. *ACS Appl. Mater. Interfaces* **2018,** *10* (19), 16650-16659.

2. Dolomanov, O.V., Bourhis, L.J., Gildea, R.J, Howard, J.A.K. & Puschmann, H. OLEX2：A

Complete Structure Solution, Refinement and Analysis Program. *J. Appl. Cryst.* **2009**, *42*,339.

3. Sheldrick, G.M. SHELXT-Integrated Space-Group and Crystal-Structure Determination. *Acta Cryst*. **2015**, A71, 3-8.

4. Sheldrick, G.M. Crystal Structure Refinement with SHELXL. *Acta Cryst*. **2015**, C71, 3-8.

5. Zhang, Y.; Zhang, X.; Lyu, J.; Otake, K. I.; Wang, X.; Redfern, L. R.; Malliakas, C. D.; Li, Z.; Islamoglu, T.; Wang, B.; Farha, O. K., A Flexible Metal-Organic Framework with 4-Connected Zr6 Nodes. *J. Am. Chem. Soc.* **2018,** *140* (36), 11179-11183.

6. Padalkar, V. S.; Seki, S., Excited-state intramolecular proton-transfer (ESIPT)-inspired solid state emitters. *Chem. Soc. Rev.* **2016,** *45* (1), 169-202.

7. Si, J.; Xia, H.-L.; Zhou, K.; Li, J.; Xing, K.; Miao, J.; Zhang, J.; Wang, H.; Qu, L.-L.; Liu, X.-Y.; Li, J., Reticular Chemistry with Art: A Case Study of Olympic Rings-Inspired Metal–Organic Frameworks. *J. Am. Chem. Soc.* **2022,** *144* (48), 22170-22177.

8. Tan, H.; Wu, X.; Weng, Y.; Lu, Y.; Huang, Z.-Z., Self-Assembled FRET Nanoprobe with Metal–Organic Framework As a Scaffold for Ratiometric Detection of Hypochlorous Acid. *Anal. Chem.* **2020,** *92* (4), 3447-3454.

9. Li, Y.-A.; Yang, S.; Li, Q.-Y.; Ma, J.-P.; Zhang, S.; Dong, Y.-B., UiO-68-ol NMOF-Based Fluorescent Sensor for Selective Detection of HClO and Its Application in Bioimaging. *Inorg. Chem.* **2017,** *56* (21), 13241-13248.

10. Zeng, Y.-N.; Zheng, H.-Q.; He, X.-H.; Cao, G.-J.; Wang, B.; Wu, K.; Lin, Z.-J., Dual-emissive metal–organic framework: a novel turn-on and ratiometric fluorescent sensor for highly efficient and specific detection of hypochlorite. *Dalton Trans.* **2020,** *49* (28), 9680-9687.

11. Li, Q.-Y.; Li, Y.-A.; Guan, Q.; Li, W.-Y.; Dong, X.-J.; Dong, Y.-B., UiO-68-PT MOF-Based Sensor and Its Mixed Matrix Membrane for Detection of HClO in Water. *Inorg. Chem.* **2019,** *58* (15), 9890-9896.

12. Ma, Y.; Xu, G.; Wei, F.; Cen, Y.; Xu, X.; Shi, M.; Cheng, X.; Chai, Y.; Sohail, M.; Hu,

Q., One-Pot Synthesis of a Magnetic, Ratiometric Fluorescent Nanoprobe by Encapsulating Fe3O4 Magnetic Nanoparticles and Dual-Emissive Rhodamine B Modified Carbon Dots in Metal–Organic Framework for Enhanced HClO Sensing. *ACS Appl. Mater. Interfaces* **2018,** *10* (24), 20801-20805.

13. Xu, H.; Cao, C.-S.; Xue, J.-Z.; Xu, Y.; Zhai, B.; Zhao, B., A Cuprous/Lanthanide-Organic Framework as the Luminescent Sensor of Hypochlorite. *Chem.-Eur. J.* **2018,** *24* (41), 10296-10299.
